# Inequality in the accumulation of diseases and medications among older adults: a longitudinal cohort study 2016–2021

**DOI:** 10.1136/bmjph-2025-003636

**Published:** 2025-12-25

**Authors:** Tran Quoc Bao Tran, Stefanie Lip, Chris McParland, Barbara I Nicholl, Jocelyn M Friday, Jim Lewsey, Daniel Mackay, Ruth Dundas, Clea Du Toit, Alan Stevenson, Jill P Pell, Frances S Mair, Sandosh Padmanabhan

**Affiliations:** 1 School of Cardiovascular and Metabolic Health, University of Glasgow, Glasgow, UK; 2 Queen Elizabeth University Hospital, Glasgow, UK; 3 School of Medicine, Dentistry and Nursing, University of Glasgow, Glasgow, UK; 4 School of Health and Wellbeing, University of Glasgow, Glasgow, UK; 5 Digital Health Validation Lab, Living Lab, University of Glasgow, Glasgow, UK

**Keywords:** Epidemiology, Public Health, Comorbidity

## Abstract

**Introduction:**

Socioeconomic deprivation is a major driver of multimorbidity (multiple long-term conditions, MLTCs) and polypharmacy in ageing populations. However, it is unclear how these inequalities evolve over time or influence early disease progression.

**Methods:**

We conducted a population-based longitudinal study of 414 746 adults aged ≥51 years in Greater Glasgow and Clyde, Scotland, using linked administrative health records. Participants were followed at three timepoints (2016, 2019, 2021), with socioeconomic status defined by Scottish Index of Multiple Deprivation (SIMD) deciles. Outcomes included prevalence and progression of MLTCs and medication burden. Zero-inflated negative binomial (ZINB) models estimated the burden of disease and prescribing, and the likelihood of remaining disease-free or prescription-free, adjusted for age and sex.

**Results:**

Multimorbidity and prescription burden increased over time across all groups. In 2021, 26.0% of individuals in the most deprived decile (SIMD1) had ≥5 conditions compared with 13.8% in the least deprived (SIMD10). From 2016 to 2021, individuals in SIMD10 were nearly 40% less likely to progress from a single condition to five or more (risk ratio=0.62 (0.60 to 0.63)) compared with SIMD1. ZINB models showed lower expected MLTC counts (incidence rate ratio (IRR)=0.59 (0.58 to 0.60)) and medication burden (IRR=0.91 (0.90 to 0.91)) in SIMD10, but also lower odds of remaining completely disease-free (OR=0.65 (0.63 to 0.68)) or prescription-free (OR=0.44 (0.41 to 0.47)). Findings were consistent in survivor-only sensitivity analyses (n=360 683).

**Conclusions:**

Socioeconomic deprivation shapes disease and treatment trajectories from the earliest stages. Individuals in deprived areas experience faster accumulation of conditions and medications. These disparities are not explained by survival differences and highlight the need for equity-focused prevention, tailored care pathways and systems that address the social complexity of multimorbidity.

**WHAT IS ALREADY KNOWN ON THIS TOPIC**

- Multimorbidity is more prevalent and develops earlier in socioeconomically deprived populations.
- Polypharmacy increases the risk of adverse outcomes in older adults with multimorbidity.
- Risk stratification and targeted care models have been promoted to manage high-need patients, but their impact on cost and equity remains uncertain.

**WHAT THIS STUDY ADDS**

- In this large population-based longitudinal study, both multimorbidity and prescription burden were strongly patterned by socioeconomic status, with consistently higher levels in the most deprived groups.
- Among individuals with only one condition, those in the most deprived areas were nearly four times more likely to develop five or more conditions than their most affluent counterparts—highlighting that deprivation influences disease progression from the earliest stages.
- More affluent individuals were more likely to be disease-free or prescription-free or require fewer treatments, despite comparable age.
- Zero-inflated models revealed that less socioeconomically deprived groups had lower average disease and medication counts, but were also more likely to be completely free of treatment or diagnoses.

**HOW THIS STUDY MIGHT AFFECT RESEARCH, PRACTICE OR POLICY**

- It reinforces the need for early, equity-oriented interventions to slow the accumulation of multimorbidity in deprived populations.
- It highlights the importance of embedding structured medication reviews into care pathways for disadvantaged older adults.
- It supports the development of person-centred care models that integrate clinical risk and social context to proactively manage disease trajectories and treatment burden.

## Introduction

 As populations age, healthcare systems globally face the growing challenge of managing multiple long-term conditions (MLTCs), or multimorbidity, typically defined as the co-occurrence of two or more chronic conditions in an individual.^1^,^4^ MLTCs disproportionately affect older adults and are associated with poorer quality of life, increased treatment complexity and rising healthcare use. Socioeconomic deprivation compounds these effects: individuals in more disadvantaged circumstances are not only more likely to develop MLTCs earlier in life but also face more complex, treatment-intensive disease trajectories.^15^,^7^

While the association between socioeconomic status (SES) and multimorbidity is well-established,^1^ less is known about how these inequalities evolve over time. Individuals in the most deprived Scottish communities develop MLTCs 10–15 years earlier than their affluent counterparts.^1^ However, whether this disparity is stable, widening or narrowing remains unclear. In addition, most studies rely on a binary definition of MLTCs (eg, ≥2 conditions), limiting the ability to track how individuals progress through higher thresholds of disease burden over time.

Recent research suggests that socioeconomic deprivation may accelerate disease accumulation, not just onset.^8 9^ Nevertheless, little is known about how this manifests across the full MLTC spectrum, including transitions from no conditions to one or more, and particularly from a single condition to several conditions. Furthermore, most prior studies have not modelled the dual processes of acquiring additional conditions and remaining disease-free. Capturing both risks is essential to understanding resilience and vulnerability in different socioeconomic groups. The role of ‘zero-inflation’—in which excess zeros in MLTC counts may reflect either true health or underdiagnosis due to limited access to care—has been largely overlooked in this context.

Polypharmacy—commonly defined as five or more prescribed medications—is another key consequence of MLTCs and poses additional challenges for patients and health systems. In Scotland, the proportion of adults prescribed ≥5 medications more than doubled between 1995 and 2010, a trend only partially attributable to population ageing.^1 10^ Older adults in the most deprived areas are more than twice as likely to be prescribed ≥10 medications than those in affluent areas,^10^ heightening the risk of adverse drug events, treatment burden and poor adherence.^11^,^14^ While some polypharmacy may be appropriate, especially in older patients, its uneven distribution raises important concerns about the equity and appropriateness of prescribing.

Despite the importance of these issues, major knowledge gaps remain. First, few longitudinal studies have examined how MLTCs accumulate across multiple thresholds in relation to SES.^8^ Second, it is unclear whether socioeconomic inequalities in multimorbidity and prescribing have persisted, narrowed or worsened over time, especially in the context of healthcare reforms and the COVID-19 pandemic. Third, existing models rarely account for zero-inflation or the probability of remaining disease-free. Finally, there is little population-level evidence on how prescription burden evolves by SES. This limits our understanding of whether current prescribing practices are reinforcing or mitigating health inequalities.

This study investigates how socioeconomic deprivation shapes the development, accumulation and progression of multimorbidity and prescription burden among older adults in Scotland over a 6-year period from 2016 to 2021. Using linked administrative data, we examine long-term condition (LTC) burden across multiple disease thresholds (0 to >5) and assess both cross-sectional and longitudinal patterns. We explore the risk of MLTC progression among individuals with a single condition and separately model the likelihood of acquiring more conditions and the probability of remaining disease-free. Prescription burden is examined in parallel. All analyses are repeated in a sensitivity cohort restricted to 2021 survivors to account for differential survival by deprivation. We hypothesise that socioeconomic inequalities in both MLTCs and prescription burden have persisted or widened, with deprived groups experiencing earlier and more intensive disease accumulation and treatment exposure.

## Methods

This study is reported as per the Strengthening the Reporting of Observational Studies in Epidemiology guideline.^15^

### Study population

**Figure 1:**
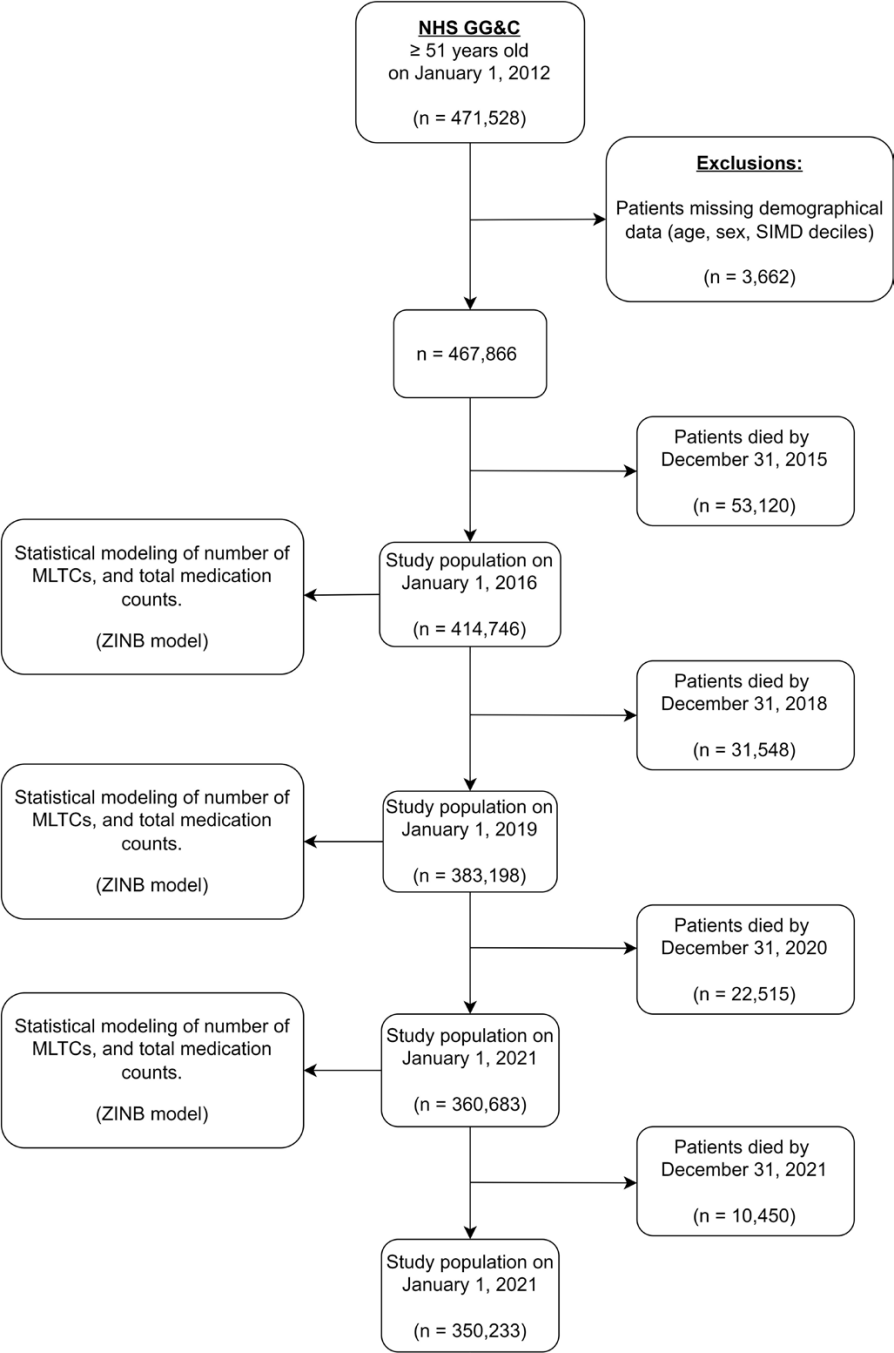
Flow diagram of the number of patients included in the analysis for the three study years 2016, 2019 and 2021. The cohort included all adults aged ≥51 years as of 1 January 2012. Patients were excluded if their demographic data, specifically age, sex or Scottish Index of Multiple Deprivation (SIMD), were missing, or they died by 31 December 2015. In 2016, the cohort comprised 414 746 adults distributed across all 10 SIMD deciles; by 2022, the cohort size decreased to 50 233, reflecting natural attrition over time. MLTCs, multiple long-term conditions; NHS GG&C, National Health Service Greater Glasgow and Clyde; ZINB, zero-inflation negative binomial.

This longitudinal cohort study used routinely collected health administrative data on patients treated in National Health Service Greater Glasgow and Clyde (NHS GG&C). NHS GG&C is the largest health board in Scotland, serving a population of approximately 1.2 million people. The West of Scotland Safe Haven, operating within NHS GG&C, is a trusted research environment in which routinely collected healthcare and administrative datasets are linked and made available for remote analysis.^16^ The cohort included all adults aged ≥51 years as of 1 January 2012. Patients were excluded if their demographic data, specifically sex, age or Scottish Index of Multiple Deprivation (SIMD), were missing, or if they died by 31 December 2015 (figure 1).

### Databases and variables

This study used linked data from three databases: The Prescribing Information System (PIS), the Scottish Morbidity Record 01 (SMR01), SMR00, Scottish Care Information (SCI) Store and death certificates. The PIS collects data on all medications dispensed in the community, which are coded in accordance with the British National Formulary (BNF). The SMR01 collects data on hospital admissions, including the date of admission, treated conditions and reason for admission, which are recorded using International Statistical Classification of Diseases and Related Health Problems 10th Revision (ICD-10) codes. The SMR00 captures episode-level data on outpatient appointments, including the date of attendance and clinic specialty. The SCI Store collates laboratory test information, capturing the date of sample collection, test type and numerical or qualitative result. Death certificates record the date and underlying cause of death also coded using ICD-10. Demographic data included the patients’ age, sex (male/female) and SIMD decile (ordinal) of the study population.

### Socioeconomic deprivation measurement

Socioeconomic deprivation was assessed using the SIMD, the official area-level measure used by the Scottish Government to quantify relative deprivation across Scotland.^17^ SIMD is purposefully constructed as a multidimensional measure of area-level deprivation that reflects cumulative disadvantage across seven domains (income, employment, health, education, housing, access to services and crime) aggregated at the level of small datazones with a mean population of ~760 residents. This aligns with the conceptual model of deprivation as a structural determinant of health, whereby the accumulation and interaction of disadvantages across domains, rather than any single domain in isolation, drives poorer outcomes. Although individual SIMD components (eg, income or education) may contribute differently across contexts, the composite index has greater explanatory power for health outcomes than single-domain measures. Moreover, SIMD is particularly suited to health service planning, as it maps geographically actionable populations and is aligned with local authority and NHS service delivery frameworks. For this study, we used the 2016 version of SIMD, and each individual in our cohort was assigned an SIMD decile (1–10) based on their area of residence, with SIMD1 representing the most deprived 10% of neighbourhoods in Scotland and SIMD10 the least deprived.

### MLTCs status assessment

MLTCs were ascertained through a combination of admission-associated ICD-10 codes in SMR01, specialty clinics attendance in SMR00, laboratory results in SCI Store and corresponding BNF-coded pharmacotherapy treatments in the PIS. The specific algorithm used to determine MLTCs is detailed in online supplemental table 1. The date of first diagnosis is defined as the earliest qualifying record in the pre-specified combination of relevant datasets (SMR01, SMR00, SCI and PIS) for each condition. The total number of MLTCs for each patient was calculated based on all conditions identified in the years up to and including the study year, representing the total number of diagnoses made prior to that year. The number of MLTCs was categorised as: 0 MLTC, 1 MLTC, 2 MLTCs, 3 MLTCs, 4 MLTCs and ≥5 MLTCs.

### Polypharmacy status assessment

For each patient, the total number of unique medications prescribed during the study year was quantified using BNF paragraph codes for different drug classes. The total number of medications was categorised as follows: 0 medications, 1 medication, 2 medications, 3–4 medications and ≥5 medications.

### Longitudinal accumulation of LTCs

We assessed changes in MLTC counts across three intervals: 2016–2018, 2019–2021 and for the full period 2016–2021. For each interval, the baseline cohort was defined on the first date (eg, 1 January 2016), and follow-up ended on the final date (eg, 31 December 2018). The outcome was the net change in the number of MLTCs (follow-up minus baseline). We excluded individuals who died during the follow-up period. This approach ensured that changes in MLTC count reflected disease accumulation only among those alive at the end of each interval.

### Statistical analyses

The demographic characteristics of the cohort were summarised using descriptive statistics. For continuous variables (eg, age) medians and IQRs were calculated. For categorical variables (eg, sex, SIMD deciles, number of MLTC categories and number of medication categories), frequencies and percentages were reported. These descriptive statistics were presented for the overall population and stratified by the three study years: 2016, 2019 and 2021. The analysis for each study year included only individuals alive on 1 January of the respective year.

We calculated the SIMD10:SIMD1 ratio for each MLTC or total medication category in 2016, 2019 and 2021, and then compared each ratio to the corresponding ratio in the reference category (‘0 MLTC’ or ‘0 medications’). This comparison provides a measure of how disparities between affluent and deprived patients vary across MLTC or medication categories relative to the baseline. The same calculations were repeated for both years, then applied to cumulative medication categories (≥2, ≥3) and MLTC categories (≥2, ≥3, ≥4, ≥5) to assess whether these disparities shifted with increasing numbers of medications or MLTCs. Finally, the calculations were repeated using ‘1 MLTC’ or ‘1 medication’ as the reference category, offering an additional perspective on how disparities in other categories compare with those in individuals who have exactly MLTC or one medication.

An additional analysis was conducted to examine how the number of patients in each MLTC or medication category changed over time. For each MLTC or medication category, the proportion of SIMD1 patients in 2021 was compared with the proportion of SIMD1 patients in 2016, and this change was then normalised against the corresponding change in the reference category (‘0 MLTC’ or ‘0 medication’). The same calculations were repeated for SIMD10 patients. This approach was also applied to the cumulative categories (≥2, ≥3, ≥4, ≥5) and again using ‘1 MLTC’ or ‘1 medication’ as the reference group, providing additional perspectives on shifts in medication use across different SES populations.

Zero-inflated negative binomial (ZINB) models were used to analyse count data characterised by overdispersion and an excess of zero counts.^18^ Zero inflation occurs when a dataset shows more zero outcomes than standard count models would predict, a phenomenon that has been underexplored in older adult populations. This excess likely reflects underlying differences in risk: some individuals may truly have no risk of the event, while others are at risk and might have zero recorded events simply by chance. While the ZINB model was chosen for its flexibility in handling count data with excess zeros, we acknowledge that other modelling approaches, such as cumulative ordinal logistic regression, could offer complementary insights. However, given the high skew, wide dispersion and the conceptual interest in the dual phenomena of accumulation and absence of multimorbidity, we judged ZINB to be the most appropriate approach for this study.

The ZINB model for assessing the association between SIMD deciles on the number of LTCs consisted of two components:

- Zero-inflation component: this component modelled the probability of an observation being an excess zero, by employing logistic regression to distinguish between individuals inherently not susceptible to MLTCs (structural zeros) and those who are susceptible.^19^ The output reflected how predictors, such as SIMD deciles, influenced the likelihood of an individual belonging to the always-zero group (ie, not susceptible to developing MLTCs).
- Count component: for individuals susceptible to LTCs, this component modelled the MLTC count using a negative binomial distribution.^20^ The results described how predictors, including SIMD deciles, were associated with the expected MLTC count.

ZINB models were adjusted for age, sex and SIMD deciles. Separate models were developed for each of the three study years (2016, 2019 and 2021). This approach was used to capture the cross-sectional distribution of disease and prescribing burden at each timepoint rather than changes within individuals over time. These timepoints reflect independent population snapshots, as individuals could enter or exit the cohort (eg, due to death or migration). Consequently, no subject-level random effects were included. While conducting analyses across multiple timepoints raises concerns of Type I error inflation, the effect sizes were highly consistent and statistically robust (p<0.001) across SIMD deciles and years. Given this consistency and the conceptual independence of cohorts at each timepoint, we report unadjusted CIs and p values, emphasising the strength and direction of effects over single-point significance testing.

ZINB models were also applied to evaluate the risk of MLTC progression over the three different time periods. Models were adjusted for age, sex, baseline MLTC count and baseline number of medications. For individuals alive at the end of each period, the ZINB model estimated (a) the expected increase in LTCs among those with any progression (count component) and (b) the odds of having no increase in LTCs (zero-inflation component).

A ZINB model was similarly applied to evaluate the association between SIMD decile and the total number of medications in each of the three study years. Covariates included in this model included age, sex, SIMD decile and number of MLTCs.

### Sensitivity analyses

To evaluate the potential impact of attrition, primarily attributable to mortality, on our findings, we conducted a series of sensitivity analyses. We restricted the cohort to participants who were alive at the end of the study period in 2021. We then repeated all descriptive analyses, recalculated risk ratios (RRs) and re-estimated models of MLTC progression within this survivor sub-cohort. This approach allowed us to determine whether the observed socioeconomic gradients persisted when the analysis was confined to individuals with complete follow-up. The resulting estimates were directly compared with those obtained from the full 2016 baseline cohort.

### Patients and public involvement

No patients or members of the public were involved in the design, conduct, reporting or dissemination of this research. This retrospective analysis relied solely on fully anonymised, routinely collected health-record data, providing no direct participant contact or scope for experiential input.

## Results

### Cohort characteristics

In 2016, the cohort comprised 414 746 adults distributed across all ten SIMD deciles; by 2021, the cohort size decreased to 360 683, reflecting natural attrition over time (figure 1). In 2016, the most deprived decile (SIMD1) patients accounted for 21.4% of the study population, and the least deprived (SIMD10) patients for 9.3%. Women comprised a slightly higher proportion (53%) overall (table 1). The mean age ranged from 69.8±12.0 years in SIMD1 to 68.7±10.1 years in SIMD10, with small but statistically significant variation across groups (p<0.001) (online supplemental table 2).

**Table 1:** Patients characteristics stratified by the three study years (2016, 2019 and 2021). Scottish Index of Multiple Deprivation (SIMD) decile 1 represents the most deprived population, and SIMD decile 10 corresponds to the most affluent

Label	Levels	2016	2019	2021	P value
Total, n (%)		414 746 (35.8)	383 198 (33.1)	360 683 (31.1)	
Age, mean (SD)		69.3 (11.2)	71.6 (10.9)	73.2 (10.9)	<0.001
Sex (%)	Female	220 748 (53.2)	203 687 (53.2)	191 646 (53.1)	0.698
	Male	193 998 (46.8)	179 511 (46.8)	169 037 (46.9)	
SIMD deciles (%)	1	88 743 (21.4)	81 195 (21.2)	75 728 (21.0)	<0.001
	2	57 969 (14.0)	52 642 (13.7)	48 939 (13.6)	
	3	36 492 (8.8)	33 341 (8.7)	31 176 (8.6)	
	4	32 050 (7.7)	29 686 (7.7)	28 064 (7.8)	
	5	29 388 (7.1)	26 899 (7.0)	25 100 (7.0)	
	6	28 036 (6.8)	26 016 (6.8)	24 460 (6.8)	
	7	25 083 (6.0)	22 918 (6.0)	21 327 (5.9)	
	8	33 603 (8.1)	31 729 (8.3)	30 432 (8.4)	
	9	44 624 (10.8)	41 970 (11.0)	40 058 (11.1)	
	10	38 758 (9.3)	36 802 (9.6)	35 399 (9.8)	
MLTC categories (%)	0 MLTC	182 615 (44.0)	153 414 (40.0)	140 965 (39.1)	<0.001
	1 MLTC	58 009 (14.0)	47 047 (12.3)	41 462 (11.5)	
	2 MLTCs	49 551 (11.9)	42 896 (11.2)	38 951 (10.8)	
	3 MLTCs	39 375 (9.5)	36 815 (9.6)	34 435 (9.5)	
	4 MLTCs	29 372 (7.1)	29 667 (7.7)	28 367 (7.9)	
	5+ MLTCs	55 824 (13.5)	73 359 (19.1)	76 503 (21.2)	
Total medications in study year—categories (%)	0 medication	133 423 (32.2)	126 146 (32.9)	124 671 (34.6)	<0.001
	1 medication	18 195 (4.4)	16 099 (4.2)	15 223 (4.2)	
	2 medications	20 015 (4.8)	17 977 (4.7)	17 441 (4.8)	
	3–4 medications	41 861 (10.1)	38 030 (9.9)	36 948 (10.2)	
	5+ medications	201 252 (48.5)	184 946 (48.3)	166 400 (46.1)	
Survival status on 1 January of the study year (%)	Alive	414 746 (100.0)	383 198 (92.4)	360 683 (87.0)	<0.001
	Deaths in previous years	0 (0.0)	31 548 (7.6)	54 063 (13.0)	

MLTCs, multiple long-term conditions.

The distribution of MLTCs varied markedly by deprivation. In 2016, 17.4% of individuals in SIMD1 had five or more MLTCs, compared with just 7.4% in SIMD10. In 2021, 26.0% of individuals in SIMD1 had five or more MLTCs, compared with just 13.8% in SIMD10. Conversely, in 2016, the proportion of individuals with no MLTCs decreased with increasing deprivation, from 49.0% in SIMD10 to 43.5% in SIMD1 (p<0.001), underscoring a clear social gradient in multimorbidity burden. The socioeconomic gradient was no longer evident in 2021, with 40.3% of individuals in SIMD10 and 41.1% in SIMD1 having no MLTC (online supplemental tables 2 and 3; online supplemental figure 1).

### Socioeconomic inequalities in multimorbidity burden and trajectories

#### Descriptive trends over time

Between 2016 and 2021, the prevalence of multimorbidity increased across all socioeconomic groups, with the greatest absolute increase in burden observed in the most deprived group. In SIMD1, the proportion of individuals who had ≥5 LTCs rose by 8.6%, from 17.4% to 26.0% (RR=1.35 (1.33 to 1.38); p<0.001) compared with an absolute increase of 6.4%, from 7.4% to 13.8%, (RR=1.95 (1.87 to 2.03); p<0.001) in SIMD10 (online supplemental tables 2–4). Although relative increases were larger in SIMD10, the absolute difference increased, and the burden remained substantially higher in SIMD1. A clear dose-response relationship was observed across all MLTC thresholds, with disease burden increasing with deprivation (online supplemental tables 2 and 3).

#### RRs across SIMD and time using MLTC=0 and MLTC=1 as reference

Online supplemental table 4 presents RRs comparing MLTC levels across socioeconomic groups and time, using two reference points: MLTC = 0 (entry into multimorbidity) and MLTC = 1 (progression beyond a single condition). In 2021, individuals in SIMD10 were significantly less likely than those in SIMD1 to reach higher MLTC thresholds. Using MLTC = 0 as reference, RRs for SIMD10 versus SIMD1 of having ≥2, ≥3, ≥4 and ≥5 MLTCs were 0.95, 0.86, 0.76 and 0.66, respectively (all p<0.001). When MLTC = 1 was the reference, RRs dropped further and were respectively 0.86, 0.79, 0.71 and 0.62 (all p<0.001). Longitudinally, between 2016 and 2021, the risk ratio(RR) of having ≥5 MLTCs increased by 35% in SIMD1 (RR 1.35 (1.33 to 1.38); p<0.001) and almost doubled in SIMD10 (RR 1.95 (1.87 to 2.03); p<0.001) when MLTC = 0 was the reference. Using MLTC = 1, these increases were 24% in SIMD1 (1.24 (1.23 to 1.26); p<0.001) and 57% in SIMD10 (1.57 (1.52 to 1.63); p<0.001). These consistent gradients across both cross-sectional and longitudinal comparisons reflect accelerated accumulation of multimorbidity across all groups, with the most deprived patients having a higher baseline burden but slower progression than the least deprived. The lower RRs observed when using MLTC = 1 as the reference suggest greater protection against further disease accumulation in more affluent groups.

#### ZINB model: MLTC count and risk of being disease-free

**Figure 2:**
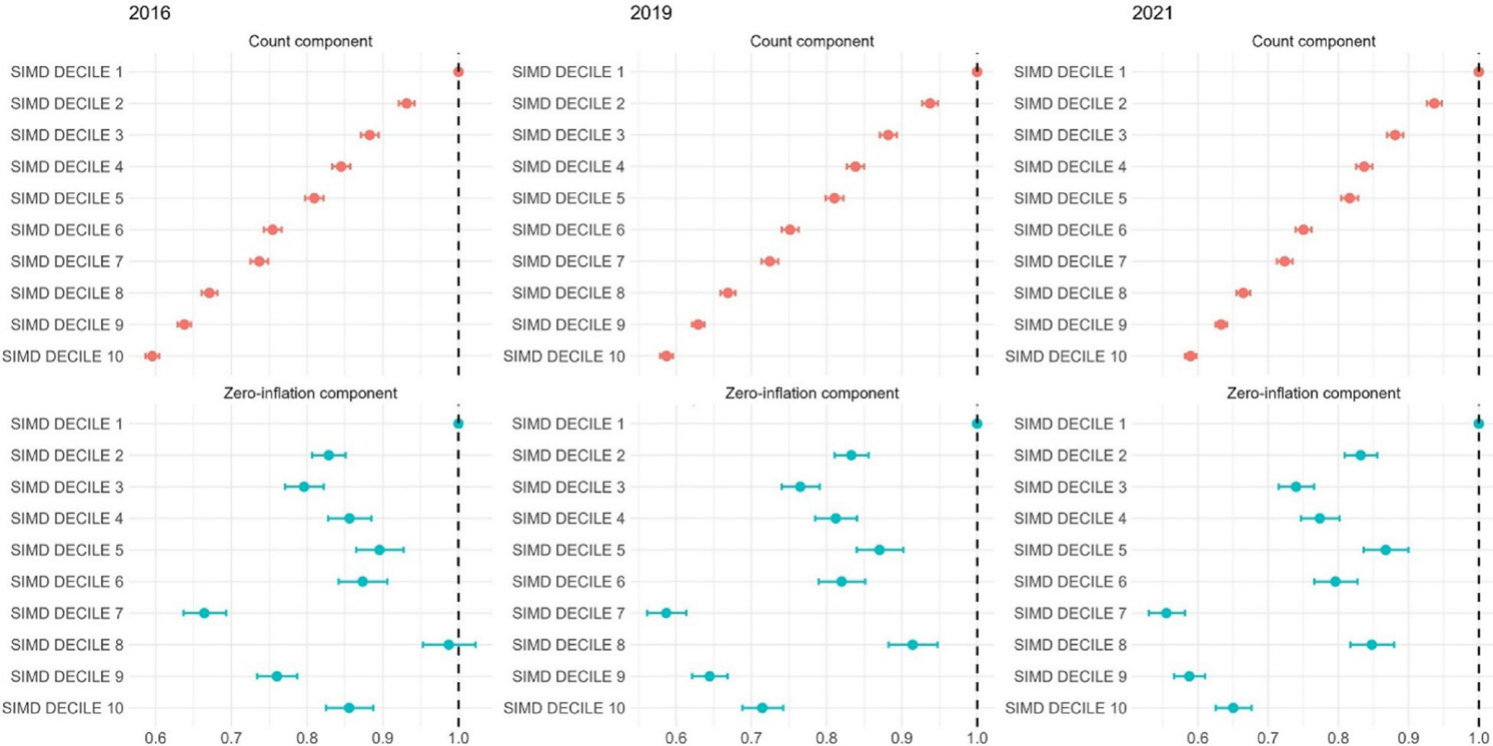
Forest plot shows results from the zero-inflated negative binomial (ZINB) model analysing count and zero-inflation components of the number of MLTCs across 10 SIMD deciles in 2016, 2019 and 2021. The count component presents incidence rate ratios (IRRs) for MLTC counts, using a negative binomial distribution, while the zero-inflation component presents ORs from logistic regression for membership in the 0 MLTC group. SIMD 1 represents the most deprived population, and SIMD 10 corresponds to the most affluent. MLTCs, multiple long-term conditions; SIMD, Scottish Index of Multiple Deprivation.

The ZINB model further clarified the distribution of multimorbidity by simultaneously estimating expected MLTC counts and the likelihood of having no conditions. The count component (incidence rate ratios (IRRs)) revealed that, in 2021, individuals in SIMD10 had 41% lower expected MLTC counts compared with SIMD1 (IRR=0.59 (0.58 to 0.60); p<0.001), with a stable and graded pattern across deciles. The zero-inflation component, modelling the odds of being disease-free, showed that those in SIMD10 had significantly lower odds of having 0 MLTC (OR=0.65 (0.63 to 0.68); *p*<0.001) compared with SIMD1 (figure 2, online supplemental table 5). These findings confirm a persistent social gradient across the full spectrum of multimorbidity: individuals in more deprived groups were more likely to accumulate multiple conditions but were also more likely to remain free of any LTCs.

#### Longitudinal accumulation of LTCs

**Figure 3:**
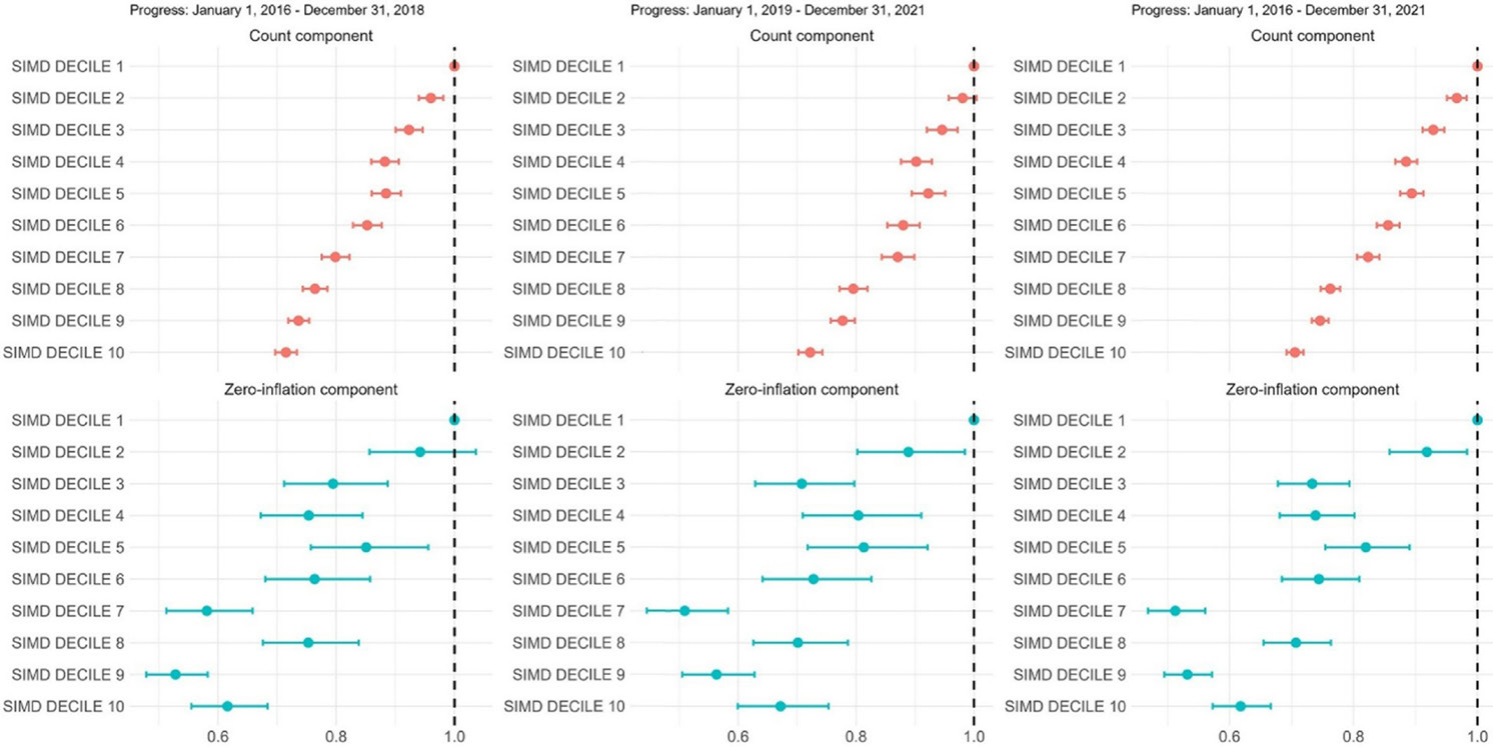
Forest plot showing results from the zero-inflated negative binomial (ZINB) model analysing count and zero-inflation components of the number of new MLTCs across 10 SIMD deciles in three intervals: 2016–2018, 2019–2021 and for the full period 2016–2021. For each interval, the baseline cohort was defined on the first date (eg, 1 January 2016), and follow-up ended on the final date (eg, 31 December 2018). The outcome was the net change in the number of MLTCs (follow-up minus baseline). The count component presents incidence rate ratios (IRRs) for new MLTC counts, using a negative binomial distribution, while the zero-inflation component presents ORs from logistic regression for membership in the zero new MLTC group. Individuals who died during a given interval were excluded. SIMD 1 represents the most deprived population, and SIMD 10 corresponds to the most affluent. MLTCs, multiple long-term conditions; SIMD, Scottish Index of Multiple Deprivation.

Among individuals who remained alive over three follow-up intervals: 2016–2018, 2019–2021 and 2016–2021, the count component of the model in each interval showed a stepwise socioeconomic gradient in the number of additional conditions accumulated, with IRRs decreasing progressively from SIMD2 to SIMD10 relative to SIMD1. For the full 2016–2021 period, individuals in SIMD10 accumulated 29% fewer new MLTCs than those in SIMD1 (IRR=0.71 (0.69 to 0.72); p<0.001). The zero-inflation component indicated that the odds of remaining stable (no new conditions) were also lower in SIMD10 compared with SIMD1 (OR=0.62 (0.57 to 0.67); p<0.001), with a graded pattern across deprivation deciles. (figure 3 and online supplemental table 6)

#### Prescription burden and socioeconomic inequality

Prescription burden followed similar patterns to multimorbidity. In 2021, 47.6% of individuals in SIMD1 were dispensed ≥5 medications compared with 40.9% in SIMD10 (RR=1.15 (1.14 to 1.16); p<0.001) (table 1). Individuals in SIMD10 were significantly more likely to receive only one medication compared with SIMD1 (RR=3.24 (3.06 to 3.44); p<0.001), and more likely to be in low-prescription categories (eg, 2–4 medications) (online supplemental table 7). When using one medication as the reference category, the RR of receiving ≥5 medications in SIMD10 was 0.90 (0.89 to 0.9); p<0.001 compared with SIMD1 (online supplemental table 7), reinforcing the concentration of higher treatment burden among the most deprived. These inequalities remained largely unchanged across time and were consistent across broader thresholds of ≥2 and ≥3 medications.

**Figure 4:**
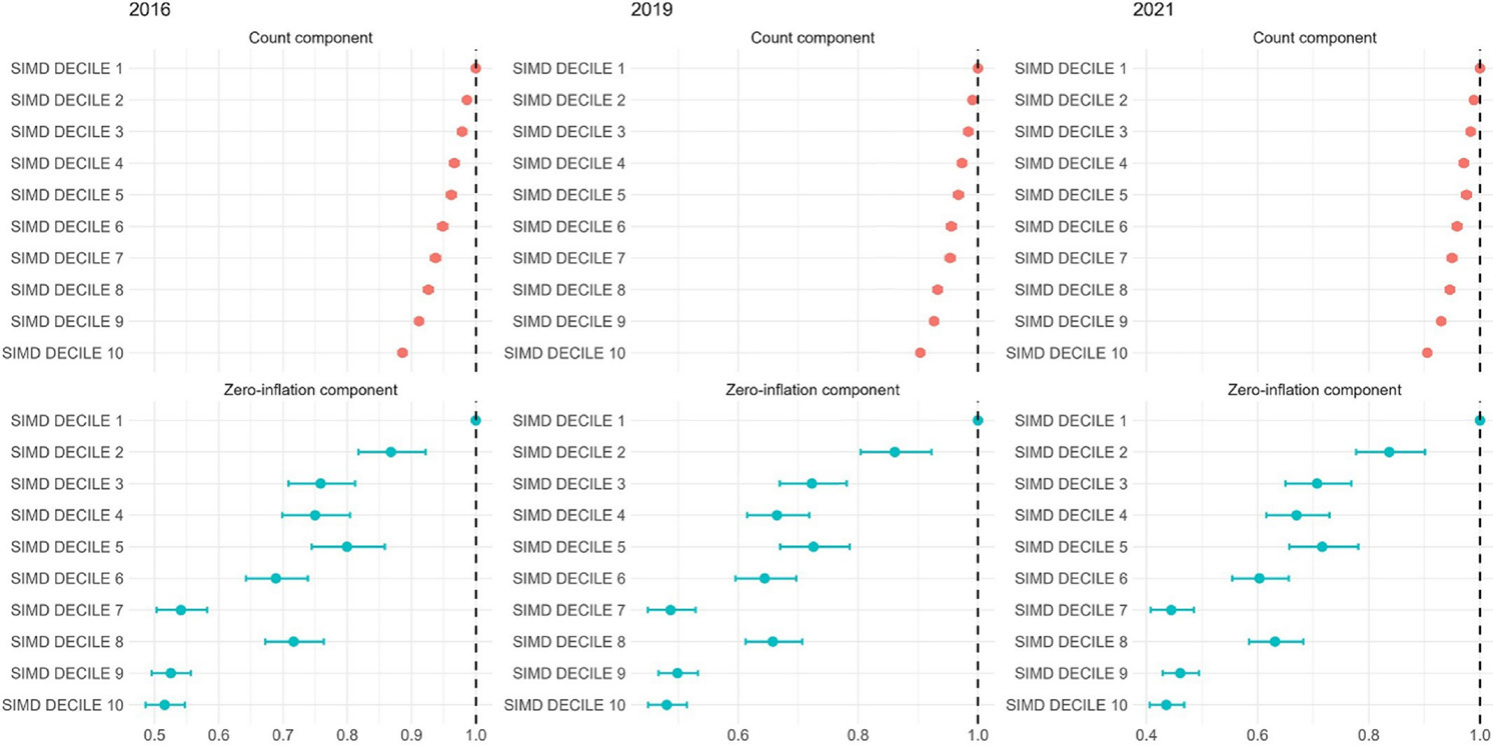
Forest plot shows results from the zero-inflated negative binomial (ZINB) model analysing count and zero-inflation components of the number of total medications across 10 SIMD deciles in 2016, 2019 and 2021. The count component presents incidence rate ratios (IRRs) for total medication counts, using a negative binomial distribution, while the zero-inflation component presents ORs from logistic regression for membership in the zero total medications group. SIMD 1 represents the most deprived population, and SIMD 10 corresponds to the most affluent. MLTCs, multiple long-term conditions; SIMD, Scottish Index of Multiple Deprivation.

ZINB models for medication count revealed that, in 2021, individuals in SIMD10 had 9.5% lower expected prescription counts than those in SIMD1 (IRR=0.90 (0.89 to 0.91); p<0.001), and were significantly less likely to be prescription-free (OR=0.44 (0.41 to 0.47); p<0.001) (figure 4, online supplemental table 8), indicating that deprivation was associated with lower likelihood of treatment exposure, but higher intensity of treatment once prescribed.

#### Sensitivity analyses

A sensitivity analysis restricted to individuals alive at the end of the follow-up period (n=360 683) confirmed the robustness of the primary findings. Across all three years (2016, 2019, 2021), the socioeconomic gradient in multimorbidity and medication burden persisted (online supplemental tables 9–14). In 2021, 21.2% of individuals had ≥5 MLTCs, up from 9.9% in 2016. Polypharmacy (≥5 medications) rose from 43.2% to 46.1%. ZINB models showed a graded decline in both the number of MLTC and the number of medications across deprivation levels, while also showing that individuals in more affluent groups had a lower probability of being completely free of disease or prescriptions (online supplemental tables 15 and 16, online supplemental figures 2 and 3).

## Discussion

This study provides a detailed, longitudinal analysis of socioeconomic inequalities in multimorbidity and prescription burden among older adults in Scotland. Using a population cohort of over 414 000 individuals across 6 years, we found a persistent and graded association between deprivation and the accumulation of LTCs and medications. These disparities spanned the entire socioeconomic gradient, with the most deprived consistently experiencing greater disease burden and treatment exposure.

Multimorbidity was both more prevalent and progressed more rapidly in socioeconomically deprived groups. By 2021, over one in four individuals in the most socioeconomically deprived decile (SIMD1) had five or more conditions, compared with 14% in the least socioeconomically deprived decile (SIMD10). Those in SIMD10 were significantly less likely to progress to higher MLTC categories, even after adjusting for baseline health and demographics. This aligns with previous findings that socioeconomic disadvantage accelerates the development and accumulation of MLTCs, potentially through cumulative exposure to risk and limited access to protective resources.^1 6 21 22^

Prescription burden mirrored these patterns. More socioeconomically deprived groups had higher rates of polypharmacy and were less likely to be prescribed only one or two medications. Our findings extend earlier work by showing that these disparities persist over time and are not fully explained by MLTC counts alone.^16 23 24^ Individuals in more affluent groups may benefit from healthier ageing trajectories, greater health literacy and more conservative or coordinated prescribing.^25 26^ However, the possibility that higher medication use in deprived areas reflects more advanced disease at presentation must be acknowledged, particularly given the lack of clinical severity data.

The results from our study also provide complementary insights into the nature of disease accumulation across socioeconomic strata. While using MLTC = 0 highlights disparities in initial entry into multimorbidity, the MLTC = 1 reference focuses attention on those already in the early stages of disease. In 2021, RRs for having five or more conditions were 34% lower in SIMD10 than SIMD1 when MLTC = 0 was the reference, but 38% lower when MLTC = 1 was used. This suggests that once a condition has developed, individuals in more affluent areas are less likely to experience further disease accumulation. These results reinforce the importance of focusing intervention efforts not only on preventing initial multimorbidity in deprived populations but also on interrupting disease progression at the early stages, particularly after the first LTC is diagnosed. Including MLTC=1 as a reference captures this critical inflection point and strengthens the rationale for early-stage, targeted prevention.

To better separate the effects of disease progression from mortality, zero-inflated models restricted to individuals who survived through follow-up revealed that individuals in less deprived groups were less likely to be completely disease-free or medication-free, despite lower average counts. This pattern may reflect differences in healthcare engagement, preventive care or diagnostic intensity, which warrants further exploration. In contrast, apparent ‘zero’ outcomes in SIMD1 may reflect underdiagnosis or lower healthcare engagement. This interpretation is supported by the concurrent finding of higher disease and prescribing counts among other individuals in SIMD1, suggesting a polarised distribution within deprived populations: some with heavy burden, and others potentially disengaged or excluded from routine care—indicating that both under-treatment and over-treatment may coexist within deprived populations, as noted in the works of Mercer and Watt^27^ and Barnett *et al*.^1^ These findings support the use of zero-inflated models in health inequalities research and caution against equating ‘zero’ across groups without considering structural differences in access and care.

These findings reinforce the concept of the inverse care law: those with the greatest need often receive the least care.^27^ In contrast, resource-rich environments often face the risk of over-prescription, as detailed in the national overprescribing review for England.^28^ Additionally, disparities in treatment burden and prescribing practices highlighted by Guthrie *et al*^29^ and Mair and May^30^ suggest that variations in healthcare delivery also contribute to these patterns. Our findings suggest that this law manifests not only in access to treatment but also in the detection and management of multimorbidity. The COVID-19 pandemic may have exacerbated these patterns, especially through service disruptions and selective survival. Sensitivity analyses restricted to survivors confirmed that observed inequalities were not artefacts of differential survival, strengthening the validity of the results.

The risk of progression from a single condition to MLTCs showed a stepwise reduction with increasing affluence. This implies that health gains may require substantial reductions in deprivation rather than marginal improvements, extending the understanding of the protective effects of affluence and the detrimental effects of deprivation.^1 11 29 31 32^ These findings support targeted investment in early-stage prevention and chronic disease management in the most deprived areas.^33^

The consistency of findings across both count-based and zero-inflated models, and across multiple MLTC thresholds, suggests that observed disparities reflect meaningful differences in disease burden and health trajectories rather than artefacts of data capture.

### Implications for policy and practice

Addressing inequalities in multimorbidity and prescribing requires a shift toward equity-oriented prevention and care that goes beyond disease management to address upstream social drivers.^4^ Our findings suggest that early-stage intervention is critical, not only for clinically high-risk individuals, but also for those facing sustained socioeconomic disadvantage, where disease accumulation can begin with a single condition and rapidly escalate. The graded nature of disease accumulation observed in this study underscores the importance of whole-population strategies. However, our results also reveal that meaningful reductions in health burden are unlikely to emerge from marginal gains alone. Health gains may require substantial improvements in socioeconomic conditions or more focused interventions beyond the most deprived strata. For policy, this points to a need for prioritised action at the earliest stages of disease trajectory—particularly among individuals with one existing LTC and at high risk of progression. Structured medication reviews, particularly in high-prescribing, high-deprivation settings, can help reduce inappropriate polypharmacy and associated risks. Embedding community-based models of care, including nurse-led reviews, social prescribing and case-finding tools, into practices serving high-deprivation areas could support earlier diagnosis and improve care continuity. Risk stratification tools that combine clinical, social and behavioural data could support earlier and more tailored interventions, although their equitable implementation remains a challenge.

While SIMD deciles offer a useful lens to detect and quantify inequality, they mask the diverse needs, assets and experiences within and across communities. Deprived areas are not monoliths. A common fallacy in population health policy is the assumption that aggregated need equates to homogeneous need. Even within the same SIMD decile, rural, urban and migrant populations may experience vastly different barriers to accessing care, engaging with services and adhering to treatment—whether due to geography, digital exclusion, stigma or prior health system mistrust.

To address these intricacies, tailored, community-informed solutions are essential. Co-production with local stakeholders (including residents with lived experience of multimorbidity, frontline practitioners and community organisations) is critical for designing interventions that are both culturally acceptable and operationally feasible. Flexible service models that can adapt to different population groups (eg, migrant workers, older carers, people with low digital literacy) are more likely to succeed in high-need settings.

We therefore advocate a reframing of intervention strategy: from area-level targeting alone to community-level co-design and contextual intelligence. This shift requires embracing ‘hyperlocal’ tailoring, not as a barrier to efficiency or scalability, but as a precondition for equitable impact. Failure to tailor risks poor uptake, wasted resources and even the widening of inequalities. Conversely, approaches rooted in community trust and insight are more likely to generate lasting benefit.

In summary, addressing inequality in ageing requires managing complexity at the intersection of biology, systems and social context and is not simply a matter of treating more diseases or prescribing better drugs. Without this recognition, health systems will remain reactive and inequitable.

### Strengths and limitations

This study draws on comprehensive, linked routinely collected data over a 6-year period, enabling analysis of real-world trends in multimorbidity and prescribing across socioeconomic groups. The use of zero-inflated models adds methodological strength by accounting for excess zeros and uncovering gradients that may otherwise be missed.

This study’s findings are highly generalisable to other high-income settings with universal healthcare systems and similar socioeconomic gradients. The use of linked administrative data and robust statistical modelling provides high internal validity. However, the study relies on hospital-coded diagnoses and lacks data from primary care, where many conditions are managed. Differences in diagnostic activity and healthcare engagement across groups may have introduced measurement bias. We also lacked data on disease severity, private care, ethnicity and prescribing appropriateness. These factors may influence both MLTC and medication counts and should be explored in future work.

A potential limitation is the use of separate ZINB models across three time points without statistical correction for multiple comparisons. However, these models were specified to reflect independent cross-sectional snapshots of a dynamically changing population rather than repeated measures of the same individuals. Given this, and the large sample sizes, consistent directionality and effect magnitude across SIMD deciles and outcomes (p<0.001), we believe the risk of Type I error is minimal. Nonetheless, this approach prioritises interpretability and temporal benchmarking over formal longitudinal inference. Future studies using linked individual-level trajectories, ordinal approaches and longitudinal mixed-effects modelling could further validate and extend these findings.

Although the SIMD provides a robust composite measure of deprivation, future work could explore whether specific domain profiles, such as access to services or crime, contribute differentially to health outcomes across geographies. However, extrapolation to younger cohorts, rural areas or other healthcare settings should be made cautiously, as disease coding, prescribing practices and healthcare access may differ. Confirmatory analyses using harmonised administrative datasets from diverse settings will be essential to test the consistency of these gradients and refine context-specific intervention strategies.

## Conclusions

Socioeconomic inequalities in multimorbidity and prescription burden are persistent, graded and detectable even at the earliest stages of disease. Individuals in more deprived areas are more likely to progress from a single condition to multiple conditions and experience higher levels of polypharmacy, while their more affluent peers are more likely to remain stable with lower treatment burden. These disparities are not explained by differential survival and reflect cumulative disadvantage across the life course. The inverse care law remains a central challenge: underdiagnosis and overtreatment coexist within deprived settings, while more affluent groups benefit from earlier detection and more measured care. Addressing these inequalities requires health systems to go beyond disease management and actively confront the social patterning of care complexity. Without such action, current models of ageing and multimorbidity care will remain inequitable and unsustainable.

## Supplementary material

10.1136/bmjph-2025-003636 online supplemental file 1

## Data Availability

Data may be obtained from a third party and are not publicly available.
