# Supplementary material for: Inequality in the accumulation of diseases and medications among older adults: a longitudinal cohort study 2016–2021
Source: BMJ Public Health. 2025 Dec 25;3(2):e003636. doi: 10.1136/bmjph-2025-003636 (PMC12742123; doi:10.1136/bmjph-2025-003636)
Supplement: online supplemental file 1 [file bmjph-3-2-s001.docx]

# Supplementary Tables

Supplementary table 1 Definition of different long-term conditions (LTCs) by admission-associated ICD-10 codes in SMR01, specialty clinics attendance in SMR00, laboratory results in SCI Store, and corresponding BNF-coded pharmacotherapy treatments in the PIS. For each condition, the date of first diagnosis is defined as the earliest qualifying record in the pre-specified combination of relevant dataset.

| Group | Condition | Inclusion criteria by either SMR01, SMR00, or SCI Store | ICD-10 codes in SMR01 | BNF-coded pharmacotherapy treatments in the PIS |
| --- | --- | --- | --- | --- |
| Cancer | Upper airway | 1st Hospitalisation in SMR01  AND  Attending oncology clinic >=1 time/year | C00-C14 |  |
| Cancer | GIT | 1st Hospitalisation in SMR01  AND  Attending oncology clinic >=1 time/year | C15-C26 |  |
| Cancer | Respiratory | 1st Hospitalisation in SMR01  AND  Attending oncology clinic >=1 time/year | C30-C39 |  |
| Cancer | Bone | 1st Hospitalisation in SMR01  AND  Attending oncology clinic >=1 time/year | C40-C41 |  |
| Cancer | Skin | 1st Hospitalisation in SMR01  AND  Attending oncology clinic >=1 time/year | C43-44 |  |
| Cancer | Mesothelial | 1st Hospitalisation in SMR01  AND  Attending oncology clinic >=1 time/year | C45-C49 |  |
| Cancer | Breast | 1st Hospitalisation in SMR01  AND  Attending oncology clinic >=1 time/year | C50 |  |
| Cancer | Female genital | 1st Hospitalisation in SMR01  AND  Attending oncology clinic >=1 time/year | C51-C58 |  |
| Cancer | Male genital | 1st Hospitalisation in SMR01  AND  Attending oncology clinic >=1 time/year | C60-C63 |  |
| Cancer | Urinary tract | 1st Hospitalisation in SMR01  AND  Attending oncology clinic >=1 time/year | C64-C68 |  |
| Cancer | CNS | 1st Hospitalisation in SMR01  AND  Attending oncology clinic >=1 time/year | C69-72 |  |
| Cancer | Thyroid/Endocrine | 1st Hospitalisation in SMR01  AND  Attending oncology clinic >=1 time/year | C73-C75 |  |
| Cancer | Neuroendocrine | 1st Hospitalisation in SMR01  AND  Attending oncology clinic >=1 time/year | C7A, C7B |  |
| Cancer | Haemological | 1st Hospitalisation in SMR01  AND  Attending oncology clinic >=1 time/year | C81-C96 |  |
| Addiction | Alcohol misuse | 1st Hospitalisation in SMR01  or  Prescription of an alcohol dependence treatment | E52, F10, G62.1, I42.6,K29.2, K70.0, K70.3, K70.9, T51, Z50.2, Z71.4, Z72.1 | **Alcohol dependence medications:**  Disulfiram: 0410010B0  Acamprosate: 0410010A0  Nalmefene: 0410010D0 |
| Blood | Anaemia | 1st Hospitalisation in SMR01 | D55-D58, D60, D61, |  |
| Blood | Coagulation disorders | 2nd Hospitalisation in SMR01 | D65-D69 |  |
| CV | Arrhythmia | 1st Hospitalisation in SMR01  OR  Commencement of antiarrhythmic medications | I44-I49, except I48 | **Antiarrhythmic medications:**  Amiodarone hydrochloride (0203020D0)  Disopyramide (0203020F0)  Disopyramide phosphate (0203020G0)  Dronedarone hydrochloride (0203020X0)  Flecainide acetate (0203020I0)  Mexiletine hydrochloride (0203020P0)  Procainamide hydrochloride (0203020S0)  Propafenone hydrochloride (0203020R0)  Quinidine sulfate (0203020U0)  Digoxin (0201010F0) |
| CV | Atrial fibrillation | 1st Hospitalisation in SMR01  OR  >= 3 concomitant dispenses of an AF medication and an oral anticoagulant | I48.0 | **AF medication:**  Bisoprolol fumarate (0204000H0)  Flecainide acetate (0203020I0)  Amiodarone hydrochloride (0203020D0)  Digoxin (0201010F0)  Dronedarone hydrochloride (0203020X0)  **Oral anticoagulants:**  Acenocoumarol (0208020H0)  Apixaban (0208020Z0)  Dabigatran etexilate (0208020X0)  Edoxaban (0208020AA)  Other oral anticoagulant preparations (020802000)  Phenindione (0208020N0)  Rivaroxaban (0208020Y0)  Warfarin sodium (0208020V0) |
| CV | Cardiomyopathy | 1st Hospitalisation in SMR01 | I42, I43 |  |
| CV | Chronic heart failure | 1st Hospitalisation in SMR01 | I50 |  |
| CV | Hypertension | Initiation of antihypertensive therapy preceding CHF/CVD | I10-I13, I15 | **Antihypertensives:**  Alpha blockers: 0205040  ACE inhibitors: 0205051  ARBs: 0205052  CCBs: 0206020  Centrally acting antihypertensives: 0205020  Loop diuretics: 0202020  K-sparing and aldosterones: 0202030  K-sparing diuretics: 0202040  Thiazides and related diuretics: 0202010  Vasodilator antihypertensives: 0205010 |
| CV | Myocardial infarction | 1st Hospitalisation in SMR01 | I21-I22 |  |
| CV | Peripheral vascular disease | 1st Hospitalisation in SMR01 | I70.2, I73 |  |
| CV | Stroke or TIA | 1st Hospitalisation in SMR01 | G45.0-G45.3, G45.8-G45.9, I60, I61, I63, I64 |  |
| CV | Congenital heart dieases | 1st Hospitalisation in SMR01 | Q20-Q28 |  |
| CV | Valvular heart disease | 1st Hospitalisation in SMR01 | I34-I37 |  |
| CV | Thromboembolism | 1st Hospitalisation in SMR01  AND  Oral anticoagulant for the next 3 months | I81-I82 | **Oral anticoagulants:**  Acenocoumarol (0208020H0)  Apixaban (0208020Z0)  Dabigatran etexilate (0208020X0)  Edoxaban (0208020AA)  Other oral anticoagulant preparations (020802000)  Phenindione (0208020N0)  Rivaroxaban (0208020Y0)  Warfarin sodium (0208020V0) |
| Endocrine | Diabetes | 1st Hospitalisation in SMR01 | E10-E14 |  |
| Endocrine | Hypothyroidism | 1st Hospitalisation in SMR01  OR  1st prescription of a hypothyroidism medication | E00–E03, E89.0 | **Hypothyroidism medications:**  Levothyroxine sodium (0602010V0)  Levothyroxine sodium and liothyronine (0602010Z0)  Liothyronine sodium (0602010M0) |
| Endocrine | Obesity | 1st Hospitalisation in SMR01 | E65-E68 |  |
| GI | Inflammatory bowel disease | (1st Hospitalisation in SMR01  AND   1st dispenses of sulfasalazine/mesalazine)  OR  prescription of sulfasalazine/mesalazine for > 3 months | K50, K51 | Balsalazide sodium (0105010D0)  Mesalazine (0105010B0)  Olsalazine sodium (0105010C0)  Sulfasalazine (0105010E0) |
| GI | Irritable bowel syndrome | 1st Hospitalisation in SMR01 | K58  Exclusion: C18-C21, C25, C56, C78.5, C79.6, D01.7, D01.9, D37.1-D37.5, K50-K51, K70.2-K70.3, K74.0, K74.2, K74.6, K86.0-K86.1, K90, K91.2 |  |
| GI | Peptic ulcer disease | >=1 hospitalisation in 2 consecutive years in SMR01  OR  1st prescription of of PPI or H2 antagonist for >= 3 months | K25.7, K25.9, K26.7, K26.9, K27.7, K27.9, K28.7, K28.9 | **Proton Pump Inhibitors:**  All BNF codes starting with 0103050  **H2 antagonist:**  All BNF codes starting with 0103010 |
| GI | Severe constipation | >=1 hospitalisation in 2 consecutive years in SMR01 | K55.8, K56.0, K56.4, K56.7, K59.0, K63.1, K63.4, K63.81, K63.88, K92.80, K92.88   Exclusion: C17-C21, C45.1, C48, C51-C58, C60-C68, C78.5-C78.6, D01.7, D01.9, D37.1-D37.5, K50-K51, K66.0, N73.6, N99.4 (K56.6 if R10.1) |  |
| Infection | Chronic viral hepatitis B | 1st Hospitalisation in SMR01  OR  Hep B surface antigen positive in SCI Store | B16, B18.0-B18.1 |  |
| Infection | Chronic viral hepatitis C | 1st Hospitalisation in SMR01  OR  Hep C RNA positive in SCI Store | B18.2 |  |
| Infection | Human immunodeficiency virus (HIV) disease | 1st Hospitalisation in SMR01  OR  HIV antibody/antigen positive   OR   Presence of lab tests for HIV antibody/antigen and CD4/CD8 multiple times in >=2 consecutive years | B20 |  |
| KUB | AKI/CKD | 1st Hospitalisation in SMR01 | N17-N19 |  |
| KUB | Glomerular/Tubular | 1st Hospitalisation in SMR01 | N00-N16 |  |
| KUB | Prostate | 1st Hospitalisation in SMR01 | N40-N42 |  |
| Liver.GB.Panc | Chronic Liver | 1st Hospitalisation in SMR01 | K70-K77 |  |
| Liver.GB.Panc | GB diseases | 1st Hospitalisation in SMR01 | K80-K83 |  |
| Liver.GB.Panc | Pancreas | 1st Hospitalisation in SMR01 | K85, K86 |  |
| MentalHealth | Intellectual disabilities | 1st Hospitalisation in SMR01 | F70-F79 |  |
| MentalHealth | Developmental | 1st Hospitalisation in SMR01 | F80-F89 |  |
| MentalHealth | Schizophrenia/Psychosis | 1st Hospitalisation in SMR01   OR  Dispenses of antipsychotics at least once per year for >=2 years | F20-F29 | **Antipsychotics:**  All BNF codes starting with 0402010 |
| MentalHealth | Anxiety | 1st Hospitalisation in SMR01   OR  Dispenses of anxiolytics at least once per year for >=2 years | F40-F48 | **Anxiolytics:**  All BNF codes starting with 0401020 |
| MentalHealth | personality | 1st Hospitalisation in SMR01 | F60-F69 |  |
| MentalHealth | Dementia | 1st Hospitalisation in SMR01 | F00–F03, F05.1, G30, G31.1 |  |
| MentalHealth | Depression | 1st Hospitalisation in SMR01   OR  Dispenses of antidepressants at least once per year for >=2 years | F20.4, F31.3–F31.5, F32, F33, F34.1, F41.2, F43.2 | **Antidepressants:**  All BNF codes starting with 04030 |
| MSK | Rheumatoid arthritis | 1st Hospitalisation in SMR01   OR  concomitant dispenses of 2 or more of hydroxychloroquine/methotrexate/NSAID/ leflunomide/sulfasalazine | M05, M06, M31.5, M32–M34, M35.1, M35.3, M36.0 | Hydroxychloroquine sulfate (1001030C0)  Methotrexate (1001030U0)  NSAID: All BNF codes starting with 1001010  Leflunomide (1001030L0)  Sulfasalazine (0105010E0) |
| MSK | Arthropathy | 1st Hospitalisation in SMR01   OR  >=1 dispense of rheumatic disease medication for > 1 year | M00-M25 | **Rheumatic disease medication:**  All BNF codes starting with 1001030 |
| MSK | Spondylopathies and dorsopathies | 1st Hospitalisation in SMR01 | M45-M49 |  |
| MSK | Osteoporosis | 1st Hospitalisation in SMR01   OR  Commencement of bisphosphonates | M80-M85 | **Bisphosphonates:**  Alendronic acid (0606020A0)  Etidronate disodium (0606020C0)  Ibandronic acid (0606020W0)  Pamidronate disodium (0606020P0)  Risedronate sodium (0606020R0)  Sodium clodronate (0606020T0)  Strontium ranelate (0606020X0)  Tiludronic acid (0606020U0)  Zoledronic acid (0606020V0) |
| Neurology | Chronic pain | 1st Hospitalisation in SMR01   OR  Opioid analgesics, or non-opioid analgesics, or treatment for neuropathic pain for at least 3 months/year for >= 3 years | F45.4, M08.1, M25.50, M25.51, M25.55 - M25.57, M43.2 - M43.6, M45, M46.1, M46.3, M46.4, M46.9, M47, M48.0, M48.1, M48.8, M48.9, M50.8, M50.9, M51, M53.1 - M53.3, M53.8, M53.9, M54, M60.8, M60.9, M63.3, M79.0 - M79.2, M79.6, M79.7, M96.1 | **Opioid analgesics:**  All BNF codes starting with 0407020  **Non-opioid analgesics:**  All BNF codes starting with 0407010  **Neuropathic pain medication:**  All BNF codes starting with 0407030 |
| Neurology | Epilepsy | 1st Hospitalisation in SMR01   OR  >=1 dispense of antiepileptic for > 1 year | G40-G41 | **Antiepileptics:**  All BNF codes starting with 0408010 |
| Neurology | Multiple sclerosis | 2 Hospitalisations in SMR01 | G35, G36, G37, H46 |  |
| Neurology | Parkinson’s disease | 1st Hospitalisation in SMR01   OR  >=1 dispense of Parkinson treatment for > 1 year | G20, G21, G22 | **Parkinson medications:**  All BNF codes starting with 0409010 or 0409020 |
| Neurology | Inflammatory | 1st Hospitalisation in SMR01 | G00-G09 |  |
| Neurology | Other degenerative diseases | 1st Hospitalisation in SMR01 | G10-G32, except G20-G22 |  |
| Neurology | Neuropathy | 1st Hospitalisation in SMR01 | G50-G65 |  |
| Neurology | Muscle | 1st Hospitalisation in SMR01 | G70-G73 |  |
| Neurology | Paralytic/palsy | 1st Hospitalisation in SMR01 | G80-G83 |  |
| Pelvic | Female pelvic diseases | 1st Hospitalisation in SMR01 | N70-N98 |  |
| Pelvic | Male genital | 1st Hospitalisation in SMR01 | N43-N53 |  |
| Respiratory | COPD/asthma | 1st Hospitalisation in SMR01 | J40-J47 |  |
| Respiratory | External agents | 1st Hospitalisation in SMR01 | J60-J70 |  |
| Respiratory | Pleural | 1st Hospitalisation in SMR01 | J90-J94 |  |
| Respiratory | Infiltrative diseases | 1st Hospitalisation in SMR01 | J82, J84 |  |
| Skin | Psoriasis | 1st Hospitalisation in SMR01   OR  >=1 dispense of psoriasis preparations | L40.0 - L40.4, L40.8, L40.9 | **Psoriasis preparations:**  All BNF codes starting with 1305020 |
| Skin | Bullous | 1st Hospitalisation in SMR01 | L10-L14 |  |
| Skin | Dermatitis | 1st Hospitalisation in SMR01 | L20-L30 |  |
| Skin | Papulosquamous | 1st Hospitalisation in SMR01 | L41-L45 |  |

*Supplementary table 2 Patients characteristics in 2016, stratified Scottish Index of Multiple Deprivation (SIMD) deciles. SIMD 1 represents the most deprived population, and SIMD10 corresponds to the most affluent. MLTC: Multiple long-term conditions, SIMD: Scottish Index of Multiple Deprivation*

| Label | Levels | SIMD 1 | SIMD 2 | SIMD 3 | SIMD 4 | SIMD 5 | SIMD 6 | SIMD 7 | SIMD 8 | SIMD 9 | SIMD 10 | p |
| --- | --- | --- | --- | --- | --- | --- | --- | --- | --- | --- | --- | --- |
| Total N (%) |  | 88,743 (21.4) | 57,969 (14.0) | 36,492 (8.8) | 32,050 (7.7) | 29,388 (7.1) | 28,036 (6.8) | 25,083 (6.0) | 33,603 (8.1) | 44,624 (10.8) | 38,758 (9.3) |  |
| Age | Mean (SD) | 69.8 (12.0) | 69.8 (11.6) | 69.6 (11.2) | 69.2 (11.1) | 69.8 (11.5) | 69.1 (10.9) | 69.5 (10.8) | 68.3 (10.4) | 68.7 (10.3) | 68.7 (10.1) | <0.001 |
| Sex (%) | Female | 47,131 (53.1) | 31,262 (53.9) | 19,748 (54.1) | 17,100 (53.4) | 15,479 (52.7) | 14,877 (53.1) | 13,629 (54.3) | 17,386 (51.7) | 23,825 (53.4) | 20,311 (52.4) | <0.001 |
|  | Male | 41,612 (46.9) | 26,707 (46.1) | 16,744 (45.9) | 14,950 (46.6) | 13,909 (47.3) | 13,159 (46.9) | 11,454 (45.7) | 16,217 (48.3) | 20,799 (46.6) | 18,447 (47.6) |  |
| MLTCs categories (%) | 0 MLTCs | 38,567 (43.5) | 23,595 (40.7) | 14,856 (40.7) | 13,692 (42.7) | 13,059 (44.4) | 12,643 (45.1) | 10,223 (40.8) | 16,592 (49.4) | 20,405 (45.7) | 18,983 (49.0) | <0.001 |
|  | 1 MLTCs | 9,618 (10.8) | 7,123 (12.3) | 4,840 (13.3) | 4,508 (14.1) | 3,951 (13.4) | 4,191 (14.9) | 4,063 (16.2) | 5,290 (15.7) | 7,756 (17.4) | 6,669 (17.2) |  |
|  | 2 MLTCs | 9,565 (10.8) | 6,791 (11.7) | 4,383 (12.0) | 3,837 (12.0) | 3,518 (12.0) | 3,382 (12.1) | 3,337 (13.3) | 4,059 (12.1) | 5,755 (12.9) | 4,924 (12.7) |  |
|  | 3 MLTCs | 8,446 (9.5) | 5,787 (10.0) | 3,709 (10.2) | 3,185 (9.9) | 2,804 (9.5) | 2,694 (9.6) | 2,474 (9.9) | 2,890 (8.6) | 4,092 (9.2) | 3,294 (8.5) |  |
|  | 4 MLTCs | 7,112 (8.0) | 4,770 (8.2) | 2,855 (7.8) | 2,329 (7.3) | 2,121 (7.2) | 1,798 (6.4) | 1,806 (7.2) | 1,859 (5.5) | 2,689 (6.0) | 2,033 (5.2) |  |
|  | 5+ MLTCs | 15,435 (17.4) | 9,903 (17.1) | 5,849 (16.0) | 4,499 (14.0) | 3,935 (13.4) | 3,328 (11.9) | 3,180 (12.7) | 2,913 (8.7) | 3,927 (8.8) | 2,855 (7.4) |  |
| Total medicines in study year – categories (%) | 0 medication | 31,368 (35.3) | 18,412 (31.8) | 11,030 (30.2) | 10,058 (31.4) | 9,873 (33.6) | 9,092 (32.4) | 6,751 (26.9) | 11,924 (35.5) | 12,999 (29.1) | 11,916 (30.7) | <0.001 |
|  | 1 medication | 2,411 (2.7) | 1,800 (3.1) | 1,377 (3.8) | 1,280 (4.0) | 1,171 (4.0) | 1,357 (4.8) | 1,341 (5.3) | 1,815 (5.4) | 2,863 (6.4) | 2,780 (7.2) |  |
|  | 2 medications | 2,729 (3.1) | 2,172 (3.7) | 1,552 (4.3) | 1,495 (4.7) | 1,300 (4.4) | 1,497 (5.3) | 1,434 (5.7) | 1,927 (5.7) | 3,076 (6.9) | 2,833 (7.3) |  |
|  | 3-4 medications | 6,537 (7.4) | 4,906 (8.5) | 3,272 (9.0) | 3,250 (10.1) | 2,845 (9.7) | 2,983 (10.6) | 3,033 (12.1) | 3,917 (11.7) | 5,792 (13.0) | 5,326 (13.7) |  |
|  | 5+ medications | 45,698 (51.5) | 30,679 (52.9) | 19,261 (52.8) | 15,967 (49.8) | 14,199 (48.3) | 13,107 (46.8) | 12,524 (49.9) | 14,020 (41.7) | 19,894 (44.6) | 15,903 (41.0) |  |

*Supplemenary table 3 Patients characteristics in 2021, stratified by SIMD deciles. SIMD 1 represents the most deprived population, and SIMD10 corresponds to the most affluent. MLTC: Multiple long-term conditions, SIMD: Scottish Index of Multiple Deprivation*

| Label | Levels | SIMD 1 | SIMD 2 | SIMD 3 | SIMD 4 | SIMD 5 | SIMD 6 | SIMD 7 | SIMD 8 | SIMD 9 | SIMD 10 | p |
| --- | --- | --- | --- | --- | --- | --- | --- | --- | --- | --- | --- | --- |
| Total N (%) |  | 75,728 (21.0) | 48,939 (13.6) | 31,176 (8.6) | 28,064 (7.8) | 25,100 (7.0) | 24,460 (6.8) | 21,327 (5.9) | 30,432 (8.4) | 40,058 (11.1) | 35,399 (9.8) |  |
| Age | Mean (SD) | 74.0 (12.1) | 73.5 (11.4) | 73.4 (11.0) | 73.2 (11.0) | 73.3 (11.1) | 72.8 (10.4) | 72.8 (10.1) | 72.4 (10.0) | 72.5 (9.6) | 72.7 (9.6) | <0.001 |
| Sex (%) | Female | 40,236 (53.1) | 26,245 (53.6) | 16,898 (54.2) | 15,026 (53.5) | 13,124 (52.3) | 12,903 (52.8) | 11,505 (53.9) | 15,754 (51.8) | 21,340 (53.3) | 18,615 (52.6) | <0.001 |
|  | Male | 35,492 (46.9) | 22,694 (46.4) | 14,278 (45.8) | 13,038 (46.5) | 11,976 (47.7) | 11,557 (47.2) | 9,822 (46.1) | 14,678 (48.2) | 18,718 (46.7) | 16,784 (47.4) |  |
| MLTCs categories (%) | 0 MLTCs | 31,158 (41.1) | 18,584 (38.0) | 11,344 (36.4) | 10,564 (37.6) | 10,162 (40.5) | 9,714 (39.7) | 7,301 (34.2) | 12,919 (42.5) | 14,946 (37.3) | 14,273 (40.3) | <0.001 |
|  | 1 MLTCs | 6,011 (7.9) | 4,468 (9.1) | 3,226 (10.3) | 3,220 (11.5) | 2,711 (10.8) | 3,034 (12.4) | 3,030 (14.2) | 4,132 (13.6) | 6,176 (15.4) | 5,454 (15.4) |  |
|  | 2 MLTCs | 6,500 (8.6) | 4,686 (9.6) | 3,266 (10.5) | 2,971 (10.6) | 2,619 (10.4) | 2,694 (11.0) | 2,688 (12.6) | 3,621 (11.9) | 5,218 (13.0) | 4,688 (13.2) |  |
|  | 3 MLTCs | 6,461 (8.5) | 4,516 (9.2) | 3,000 (9.6) | 2,748 (9.8) | 2,313 (9.2) | 2,419 (9.9) | 2,260 (10.6) | 2,935 (9.6) | 4,208 (10.5) | 3,575 (10.1) |  |
|  | 4 MLTCs | 5,915 (7.8) | 3,965 (8.1) | 2,628 (8.4) | 2,281 (8.1) | 2,036 (8.1) | 1,953 (8.0) | 1,796 (8.4) | 2,101 (6.9) | 3,161 (7.9) | 2,531 (7.1) |  |
|  | 5+ MLTCs | 19,683 (26.0) | 12,720 (26.0) | 7,712 (24.7) | 6,280 (22.4) | 5,259 (21.0) | 4,646 (19.0) | 4,252 (19.9) | 4,724 (15.5) | 6,349 (15.8) | 4,878 (13.8) |  |
| Total medicines in study year – categories (%) | 0 medication | 30,194 (39.9) | 17,453 (35.7) | 10,273 (33.0) | 9,418 (33.6) | 9,144 (36.4) | 8,435 (34.5) | 5,949 (27.9) | 11,053 (36.3) | 11,775 (29.4) | 10,977 (31.0) | <0.001 |
|  | 1 medication | 1,784 (2.4) | 1,422 (2.9) | 1,124 (3.6) | 1,126 (4.0) | 975 (3.9) | 1,122 (4.6) | 1,165 (5.5) | 1,589 (5.2) | 2,490 (6.2) | 2,426 (6.9) |  |
|  | 2 medications | 2,267 (3.0) | 1,748 (3.6) | 1,287 (4.1) | 1,277 (4.6) | 1,105 (4.4) | 1,282 (5.2) | 1,367 (6.4) | 1,800 (5.9) | 2,733 (6.8) | 2,575 (7.3) |  |
|  | 3-4 medications | 5,413 (7.1) | 4,138 (8.5) | 2,851 (9.1) | 2,809 (10.0) | 2,497 (9.9) | 2,676 (109) | 2,631 (12.3) | 3,586 (11.8) | 5,388 (13.5) | 4,959 (14.0) |  |
|  | 5+ medications | 36,070 (47.6) | 24,178 (49.4) | 15,641 (50.2) | 13,434 (47.9) | 11,379 (45.3) | 10,945 (44.7) | 10,215 (47.9) | 12,404 (40.8) | 17,672 (44.1) | 14,462 (40.9) |  |

Supplementary table 4 Comparison of SIMD10 and SIMD1 risk ratios across multiple long-term condition (MLTCs) categories in 2016 and 2021, using 0 or 1 MLTCs as reference groups. RR: risk ration, SIMD: Scottish Index of Multiple Deprivation.

|  | Categories of MLTCs number | 2016 SIMD10 vs SIMD1  RR (95% CI) | 2021 SIMD10 vs SIMD1  RR (95% CI) | SIMD1 2021 vs 2016  RR (95% CI) | SIMD10 2021 vs 2016  RR (95% CI) |
| --- | --- | --- | --- | --- | --- |
| Using zero MLTCs as the reference category | 0 MLTCs | 1 | 1 | 1 | 1 |
|  | 1 MLTCs | 1.3 [1.27, 1.34]; p < 0.001 | 1.71 [1.66, 1.77]; p < 0.001 | 0.81 [0.79, 0.83]; p < 0.001 | 1.06 [1.03, 1.1]; p < 0.001 |
|  | 2 MLTCs | 1.04 [1.01, 1.07]; p = 0.022 | 1.43 [1.39, 1.48]; p < 0.001 | 0.87 [0.84, 0.89]; p < 0.001 | 1.2 [1.16, 1.24]; p < 0.001 |
|  | 3 MLTCs | 0.82 [0.79, 0.85]; p < 0.001 | 1.17 [1.12, 1.21]; p < 0.001 | 0.96 [0.93, 0.98]; p = 0.003 | 1.35 [1.3, 1.41]; p < 0.001 |
|  | 4 MLTCs | 0.62 [0.59, 0.65]; p < 0.001 | 0.94 [0.9, 0.99]; p = 0.008 | 1.02 [0.99, 1.06]; p = 0.13 | 1.56 [1.47, 1.64]; p < 0.001 |
|  | 5+ MLTCs | 0.46 [0.44, 0.47]; p < 0.001 | 0.66 [0.64, 0.68]; p < 0.001 | 1.35 [1.33, 1.38]; p < 0.001 | 1.95 [1.87, 2.03]; p < 0.001 |
| Using zero MLTCs as the reference category  (Cumulative) | 0 MLTCs | 1 | 1 | 1 | 1 |
|  | 1+ MLTCs | 0.9 [0.89, 0.91]; p < 0.001 | 1.01 [1, 1.02]; p = 0.009 | 1.04 [1.03, 1.05]; p < 0.001 | 1.17 [1.15, 1.18]; p < 0.001 |
|  | 2+ MLTCs | 0.8 [0.79, 0.81]; p < 0.001 | 0.95 [0.93, 0.96]; p < 0.001 | 1.08 [1.07, 1.09]; p < 0.001 | 1.28 [1.26, 1.3]; p < 0.001 |
|  | 3+ MLTCs | 0.68 [0.66, 0.69]; p < 0.001 | 0.86 [0.84, 0.87]; p < 0.001 | 1.14 [1.13, 1.15]; p < 0.001 | 1.44 [1.41, 1.48]; p < 0.001 |
|  | 4+ MLTCs | 0.56 [0.54, 0.57]; p < 0.001 | 0.76 [0.74, 0.77]; p < 0.001 | 1.22 [1.21, 1.24]; p < 0.001 | 1.67 [1.62, 1.72]; p < 0.001 |
|  | 5+ MLTCs | 0.46 [0.44, 0.47]; p < 0.001 | 0.66 [0.64, 0.68]; p < 0.001 | 1.35 [1.33, 1.38]; p < 0.001 | 1.95 [1.87, 2.03]; p < 0.001 |
| Using one MLTCs as the reference category | 1 MLTCs | 1 | 1 | 1 | 1 |
|  | 2 MLTCs | 0.85 [0.83, 0.87]; p < 0.001 | 0.89 [0.87, 0.91]; p < 0.001 | 1.04 [1.02, 1.07]; p < 0.001 | 1.09 [1.06, 1.12]; p < 0.001 |
|  | 3 MLTCs | 0.71 [0.68, 0.73]; p < 0.001 | 0.76 [0.74, 0.79]; p < 0.001 | 1.11 [1.08, 1.13]; p < 0.001 | 1.2 [1.15, 1.24]; p < 0.001 |
|  | 4 MLTCs | 0.55 [0.53, 0.57]; p < 0.001 | 0.64 [0.62, 0.66]; p < 0.001 | 1.17 [1.14, 1.2]; p < 0.001 | 1.36 [1.29, 1.43]; p < 0.001 |
|  | 5+ MLTCs | 0.49 [0.47, 0.5]; p < 0.001 | 0.62 [0.60, 0.63]; p < 0.001 | 1.24 [1.23, 1.26]; p < 0.001 | 1.57 [1.52, 1.63]; p < 0.001 |
| Using one MLTCs as the reference category  (Cumulative) | 1 MLTCs | 1 | 1 | 1 | 1 |
|  | 2+ MLTCs | 0.82 [0.81, 0.83]; p < 0.001 | 0.86 [0.85, 0.87]; p < 0.001 | 1.07 [1.06, 1.08]; p < 0.001 | 1.12 [1.11, 1.13]; p < 0.001 |
|  | 3+ MLTCs | 0.72 [0.71, 0.73]; p < 0.001 | 0.79 [0.78, 0.8]; p < 0.001 | 1.1 [1.1, 1.11]; p < 0.001 | 1.21 [1.19, 1.23]; p < 0.001 |
|  | 4+ MLTCs | 0.6 [0.59, 0.62]; p < 0.001 | 0.71 [0.7, 0.72]; p < 0.001 | 1.16 [1.15, 1.17]; p < 0.001 | 1.36 [1.33, 1.4]; p < 0.001 |
|  | 5+ MLTCs | 0.49 [0.47, 0.5]; p < 0.001 | 0.62 [0.6, 0.63]; p < 0.001 | 1.24 [1.23, 1.26]; p < 0.001 | 1.57 [1.52, 1.63]; p < 0.001 |

Supplementary table 5 Results from the Zero-Inflated Negative Binomial (ZINB) model analyzing count and zero-inflation components of MLTCs across 10 SIMD deciles in 2016, 2019, and 2021. The count component columns present incidence rate ratios (IRR) for MLTCs counts, using a negative binomial distribution, while the zero-inflation component columns present odds ratios (OR) from logistic regression for membership in the zero MLTCs group. SIMD 1 represents the most deprived population, and SIMD 10 corresponds to the most affluent. IRR: incidence rate ratio, OR: odd ratio, SIMD: Scottish Index of Multiple Deprivation.

| SIMD | Count component – 2016  IRR (95% CI) | Count component – 2019  IRR (95% CI) | Count component – 2021  IRR (95% CI) | Zero Inflation component – 2016  OR (95% CI) | Zero Inflation component – 2019  OR (95% CI) | Zero Inflation component – 2021  OR (95% CI) |
| --- | --- | --- | --- | --- | --- | --- |
| SIMD 1 | 1 | 1 | 1 | 1 | 1 | 1 |
| SIMD 2 | 0.93 [0.92 - 0.94] ; P value <0.001 | 0.94 [0.93 - 0.95]; P value <0.001 | 0.94 [0.93 - 0.95] ; P value <0.001 | 0.83 [0.81 - 0.85] ; P value <0.001 | 0.83 [0.81 - 0.86] ; P value <0.001 | 0.83 [0.81 - 0.86] ; P value <0.001 |
| SIMD 3 | 0.88 [0.87 - 0.89] ; P value <0.001 | 0.88 [0.87 - 0.89] ; P value <0.001 | 0.88 [0.87 - 0.89] ; P value <0.001 | 0.80 [0.77 - 0.82] ; P value <0.001 | 0.77 [0.74 - 0.79] ; P value <0.001 | 0.74 [0.72 - 0.77] ; P value <0.001 |
| SIMD 4 | 0.85 [0.83 - 0.86] ; P value <0.001 | 0.84 [0.83 - 0.85] ; P value <0.001 | 0.84 [0.83 - 0.85] ; P value <0.001 | 0.86 [0.83 - 0.89] ; P value <0.001 | 0.81 [0.79 - 0.84] ; P value <0.001 | 0.77 [0.75 - 0.80] ; P value <0.001 |
| SIMD 5 | 0.81 [0.80 - 0.82] ; P value <0.001 | 0.81 [0.80 - 0.82] ; P value <0.001 | 0.82 [0.80 - 0.83] ; P value <0.001 | 0.90 [0.87 - 0.93] ; P value <0.001 | 0.87 [0.84 - 0.90] ; P value <0.001 | 0.87 [0.84 - 0.90] ; P value <0.001 |
| SIMD 6 | 0.75 [0.74 - 0.77] ; P value <0.001 | 0.75 [0.74 - 0.76] ; P value <0.001 | 0.75 [0.74 - 0.76] ; P value <0.001 | 0.87 [0.84 - 0.91] ; P value <0.001 | 0.82 [0.79 - 0.85] ; P value <0.001 | 0.80 [0.77 - 0.83] ; P value <0.001 |
| SIMD 7 | 0.74 [0.73 - 0.75] ; P value <0.001 | 0.72 [0.71 - 0.74] ; P value <0.001 | 0.72 [0.71 - 0.74] ; P value <0.001 | 0.66 [0.64 - 0.69] ; P value <0.001 | 0.59 [0.56 - 0.61] ; P value <0.001 | 0.56 [0.53 - 0.58] ; P value <0.001 |
| SIMD 8 | 0.67 [0.66 - 0.68] ; P value <0.001 | 0.67 [0.66 - 0.68] ; P value <0.001 | 0.67 [0.66 - 0.67] ; P value <0.001 | 0.99 [0.95 - 1.02] ; P value <0.001 | 0.91 [0.88 - 0.95] ; P value <0.001 | 0.85 [0.82 - 0.88] ; P value <0.001 |
| SIMD 9 | 0.64 [0.63 - 0.65] ; P value <0.001 | 0.63 [0.62 - 0.64] ; P value <0.001 | 0.63 [0.63 - 0.64] ; P value <0.001 | 0.76 [0.73 - 0.79] ; P value <0.001 | 0.64 [0.62 - 0.67] ; P value <0.001 | 0.59 [0.57 - 0.61] ; P value <0.001 |
| SIMD 10 | 0.60 [0.59 - 0.61] ; P value <0.001 | 0.59 [0.58 - 0.60] ; P value <0.001 | 0.59 [0.58 - 0.60] ; P value <0.001 | 0.86 [0.83 - 0.89] ; P value <0.001 | 0.71 [0.69 - 0.74] ; P value <0.001 | 0.65 [0.63 - 0.68] ; P value <0.001 |

Supplementary table 6 Results from the Zero-Inflated Negative Binomial (ZINB) model analyzing count and zero-inflation components of of the number of new MLTCs across 10 SIMD deciles in three intervals: 2016–2018, 2019–2021, and for the full period 2016–2021. For each interval, the baseline cohort was defined on the first date (e.g., 01/01/2016), and follow-up ended on the final date (e.g., 31/12/2018). The outcome was the net change in the number of MLTCs (follow-up minus baseline). The count component presents incidence rate ratios (IRR) for new MLTCs counts, using a negative binomial distribution, while the zero-inflation component presents odds ratios (OR) from logistic regression for membership in the zero new MLTCs group. Individuals who died during a given interval were excluded. SIMD 1 represents the most deprived population, and SIMD 10 corresponds to the most affluent. IRR: incidence rate ratio, OR: odd ratio, SIMD: Scottish Index of Multiple Deprivation.

| SIMD | Count component –2016-2018  IRR (95% CI) | Count component –2019-2021  IRR (95% CI) | Count component –2016-2021  IRR (95% CI) | Zero Inflation component –  2016-2018  OR (95% CI) | Zero Inflation component –  2019-2021  OR (95% CI) | Zero Inflation component –  2016-2021  OR (95% CI) |
| --- | --- | --- | --- | --- | --- | --- |
| SIMD 1 | 1 | 1 | 1 | 1 | 1 | 1 |
| SIMD 2 | 0.96 [0.94 - 0.98] ; P value <0.001 | 0.98 [0.96 - 1.00] ; P value =0.110 | 0.97 [0.95 - 0.98] ; P value <0.001 | 0.94 [0.86 - 1.03] ; P value = 0.218 | 0.89 [0.80 - 0.98] ; P value =0.024 | 0.92 [0.86 - 0.88] ; P value = 0.014 |
| SIMD 3 | 0.92 [0.90 - 0.95] ; P value <0.001 | 0.95 [0.92 - 0.97] ; P value <0.001 | 0.93 [0.91 - 0.95] ; P value <0.001 | 0.79 [0.71- 0.89] ; P value <0.001 | 0.71 [0.63 - 0.80] ; P value <0.001 | 0.73 [0.68- 0.79] ; P value <0.001 |
| SIMD 4 | 0.88 [0.86 - 0.90] ; P value <0.001 | 0.90 [0.87 - 0.93] ; P value <0.001 | 0.88 [0.86 - 0.90] ; P value <0.001 | 0.75 [0.67 - 0.84] ; P value <0.001 | 0.80 [0.71 - 0.91] ; P value <0.001 | 0.74 [0.68 - 0.80] ; P value <0.001 |
| SIMD 5 | 0.88 [0.86 - 0.91] ; P value <0.001 | 0.92 [0.89 - 0.95] ; P value <0.001 | 0.89 [0.87 - 0.91] ; P value <0.001 | 0.85 [0.76 - 0.95] ; P value = 0.006 | 0.81 [0.71 - 0.92] ; P value =0.001 | 0.82 [0.75 - 0.89] ; P value <0.001 |
| SIMD 6 | 0.85 [0.83 - 0.88] ; P value <0.001 | 0.88 [0.85 - 0.91] ; P value <0.001 | 0.85 [0.83 - 0.87] ; P value <0.001 | 0.76 [0.68 - 0.85] ; P value <0.001 | 0.73 [0.64 - 0.82] ; P value <0.001 | 0.74 [0.68 - 0.81] ; P value <0.001 |
| SIMD 7 | 0.80 [0.78 - 0.82] ; P value <0.001 | 0.87 [0.84 - 0.90] ; P value <0.001 | 0.82 [0.80 - 0.84] ; P value <0.001 | 0.58 [0.51 - 0.66] ; P value <0.001 | 0.51 [0.44 - 0.58] ; P value <0.001 | 0.51 [0.47 - 0.56] ; P value <0.001 |
| SIMD 8 | 0.76 [0.74 - 0.79] ; P value <0.001 | 0.80 [0.77 - 0.82] ; P value <0.001 | 0.76 [0.74 - 0.78] ; P value <0.001 | 0.75 [0.68 - 0.73] ; P value <0.001 | 0.70 [0.62 - 0.79] ; P value <0.001 | 0.71 [0.65 - 0.76] ; P value <0.001 |
| SIMD 9 | 0.74 [0.72 - 0.75] ; P value <0.001 | 0.78 [0.76 - 0.80] ; P value <0.001 | 0.75 [0.73 - 0.76] ; P value <0.001 | 0.53 [0.48 - 0.58] ; P value <0.001 | 0.56 [0.50 - 0.63] ; P value <0.001 | 0.53 [0.49 - 0.57] ; P value <0.001 |
| SIMD 10 | 0.71 [0.70 - 0.73] ; P value <0.001 | 0.72 [0.70 - 0.74] ; P value <0.001 | 0.71 [0.69 - 0.72] ; P value <0.001 | 0.62 [0.56 - 0.68] ; P value <0.001 | 0.67 [0.60 - 0.75] ; P value <0.001 | 0.62 [0.57 - 0.67] ; P value <0.001 |

Supplementary table 7 Comparison of SIMD10 and SIMD1 risk ratios across multiple medication number categories in 2016 and 2021, using 0 or 1 medication as reference groups. RR: risk ration, SIMD: Scottish Index of Multiple Deprivation.

|  | Categories of medication number | 2016 SIMD10 vs SIMD1  RR (95% CI) | 2021 SIMD10 vs SIMD1  RR (95% CI) | SIMD1 2021 vs 2016  RR (95% CI) | SIMD10 2021 vs 2016  RR (95% CI) |
| --- | --- | --- | --- | --- | --- |
| Using zero medication as the reference category | 0 medication | 1 | 1 | 1 | 1 |
|  | 1 medication | 2.65 [2.52, 2.79]; p < 0.001 | 3.24 [3.06, 3.44]; p < 0.001 | 0.78 [0.74, 0.83]; p < 0.001 | 0.96 [0.91, 1.01]; p = 0.079 |
|  | 2 medications | 2.4 [2.29, 2.52]; p < 0.001 | 2.72 [2.58, 2.87]; p < 0.001 | 0.87 [0.83, 0.92]; p < 0.001 | 0.99 [0.94, 1.04]; p = 0.658 |
|  | 3-4 medications | 1.79 [1.74, 1.85]; p < 0.001 | 2.05 [1.98, 2.12]; p < 0.001 | 0.88 [0.85, 0.91]; p < 0.001 | 1.01 [0.98, 1.04]; p = 0.653 |
|  | 5+ medications | 0.96 [0.95, 0.98]; p < 0.001 | 1.15 [1.14, 1.16]; p < 0.001 | 0.93 [0.92, 0.94]; p < 0.001 | 0.99 [0.98, 1.01]; p = 0.462 |
| Using zero medication as the reference category  (Cumulative) | 0 medication | 1 | 1 | 1 | 1 |
|  | 2+ medications | 1.05 [1.04, 1.06]; p < 0.001 | 1.13 [1.12, 1.14]; p < 0.001 | 0.93 [0.92, 0.94]; p < 0.001 | 1 [0.99, 1.01]; p = 0.635 |
|  | 3+ medications | 1.03 [1.02, 1.04]; p < 0.001 | 1.1 [1.09, 1.12]; p < 0.001 | 0.93 [0.92, 0.93]; p < 0.001 | 1 [0.99, 1.01]; p = 0.675 |
| Using one medication as the reference category | 1 medication | 1 | 1 | 1 | 1 |
|  | 2 medications | 0.95 [0.92, 0.99]; p = 0.007 | 0.92 [0.89, 0.96]; p < 0.001 | 1.05 [1.02, 1.09]; p = 0.006 | 1.02 [0.98, 1.06]; p = 0.295 |
|  | 3-4 medications | 0.9 [0.88, 0.92]; p < 0.001 | 0.89 [0.87, 0.91]; p < 0.001 | 1.03 [1.01, 1.05]; p = 0.002 | 1.02 [1.00, 1.05]; p = 0.057 |
|  | 5+ medications | 0.9 [0.89, 0.9]; p < 0.001 | 0.90 [0.89, 0.9]; p < 0.001 | 1.00 [1.00, 1.01]; p = 0.044 | 1.00 [1.00, 1.01]; p = 0.113 |
| Using one medication as the reference category  (Cumulative) | 1 medication | 1 | 1 | 1 | 1 |
|  | 2+ medications | 0.94 [0.93, 0.94]; p < 0.001 | 0.94 [0.93, 0.94]; p < 0.001 | 1.00 [1.00, 1.01]; p = 0.022 | 1.00 [1.00, 1.01]; p = 0.113 |
|  | 3+ medications | 0.93 [0.92, 0.93]; p < 0.001 | 0.93 [0.92, 0.93]; p < 0.001 | 1.00 [1.00, 1.01]; p = 0.027 | 1.01 [1.00, 1.01]; p = 0.11 |

Supplementary table 8 Results from the Zero-Inflated Negative Binomial (ZINB) model analyzing count and zero-inflation components of total medications across 10 SIMD deciles in 2016, 2019, and 2021. The count component columns present incidence rate ratios (IRR) for total medication counts, using a negative binomial distribution, while the zero-inflation component columns present odds ratios (OR) from logistic regression for membership in the zero total medication group. SIMD 1 represents the most deprived population, and SIMD 10 corresponds to the most affluent. IRR: incidence rate ratio, OR: odd ratio, SIMD: Scottish Index of Multiple Deprivation.

| SIMD | Count component – 2016  IRR (95% CI) | Count component – 2019  IRR (95% CI) | Count component – 2021  IRR (95% CI) | Zero Inflation component – 2016  OR (95% CI) | Zero Inflation component – 2019  OR (95% CI) | Zero Inflation component – 2021  OR (95% CI) |
| --- | --- | --- | --- | --- | --- | --- |
| SIMD 1 | 1 | 1 | 1 | 1 | 1 | 1 |
| SIMD 2 | 0.99 [0.98 - 0.99] ; P value <0.001 | 0.99 [0.99 - 1] ; P value 0.002 | 0.99 [0.98 - 1] ; P value <0.001 | 0.87 [0.82 - 0.92] ; P value <0.001 | 0.86 [0.8 - 0.92] ; P value <0.001 | 0.84 [0.78 - 0.9] ; P value <0.001 |
| SIMD 3 | 0.98 [0.97 - 0.99] ; P value <0.001 | 0.98 [0.98 - 0.99] ; P value <0.001 | 0.98 [0.98 - 0.99] ; P value <0.001 | 0.76 [0.71 - 0.81] ; P value <0.001 | 0.72 [0.67 - 0.78] ; P value <0.001 | 0.71 [0.65 - 0.77] ; P value <0.001 |
| SIMD 4 | 0.97 [0.96 - 0.97] ; P value <0.001 | 0.97 [0.97 - 0.98] ; P value <0.001 | 0.97 [0.96 - 0.98] ; P value <0.001 | 0.75 [0.7 - 0.8] ; P value <0.001 | 0.67 [0.62 - 0.72] ; P value <0.001 | 0.67 [0.62 - 0.73] ; P value <0.001 |
| SIMD 5 | 0.96 [0.96 - 0.97] ; P value <0.001 | 0.97 [0.96 - 0.98] ; P value <0.001 | 0.98 [0.97 - 0.98] ; P value <0.001 | 0.8 [0.74 - 0.86] ; P value <0.001 | 0.73 [0.67 - 0.79] ; P value <0.001 | 0.72 [0.66 - 0.78] ; P value <0.001 |
| SIMD 6 | 0.95 [0.94 - 0.96] ; P value <0.001 | 0.96 [0.95 - 0.96] ; P value <0.001 | 0.96 [0.95 - 0.97] ; P value <0.001 | 0.69 [0.64 - 0.74] ; P value <0.001 | 0.64 [0.6 - 0.7] ; P value <0.001 | 0.6 [0.55 - 0.66] ; P value <0.001 |
| SIMD 7 | 0.94 [0.93 - 0.95] ; P value <0.001 | 0.95 [0.95 - 0.96] ; P value <0.001 | 0.95 [0.94 - 0.96] ; P value <0.001 | 0.54 [0.5 - 0.58] ; P value <0.001 | 0.49 [0.45 - 0.53] ; P value <0.001 | 0.45 [0.41 - 0.49] ; P value <0.001 |
| SIMD 8 | 0.93 [0.92 - 0.93] ; P value <0.001 | 0.93 [0.93 - 0.94] ; P value <0.001 | 0.95 [0.94 - 0.95] ; P value <0.001 | 0.72 [0.67 - 0.76] ; P value <0.001 | 0.66 [0.61 - 0.71] ; P value <0.001 | 0.63 [0.59 - 0.68] ; P value <0.001 |
| SIMD 9 | 0.91 [0.91 - 0.92] ; P value <0.001 | 0.93 [0.92 - 0.93] ; P value <0.001 | 0.93 [0.92 - 0.94] ; P value <0.001 | 0.53 [0.5 - 0.56] ; P value <0.001 | 0.5 [0.47 - 0.53] ; P value <0.001 | 0.46 [0.43 - 0.5] ; P value <0.001 |
| SIMD 10 | 0.89 [0.88 - 0.89] ; P value <0.001 | 0.9 [0.9 - 0.91] ; P value <0.001 | 0.90 [0.89 - 0.91] ; P value <0.001 | 0.52 [0.49 - 0.55] ; P value <0.001 | 0.48 [0.45 - 0.51] ; P value <0.001 | 0.44 [0.41 - 0.47] ; P value <0.001 |

Supplementary table 9 Sensitivity analysis for patients characteristics stratified by the three study years (2016, 2019 and 2021). The cohort was restricted to participants who were alive at the end of the study period in 2021. Scottish Index of Multiple Deprivation decile 1 represents the most deprived population, and SIMD decile 10 corresponds to the most affluent. MLTCs: Multiple long-term conditions, SIMD: Scottish Index of Multiple Deprivation

| label | levels | 2016 | 2019 | 2021 | P |
| --- | --- | --- | --- | --- | --- |
| Total N (%) |  | 360,683 (33.3) | 360,683 (33.3) | 360,683 (33.3) |  |
| Age | Mean (SD) | 68.2 (10.9) | 71.2 (10.9) | 73.2 (10.9) | <0.001 |
| Sex | Female | 191,646 (53.1) | 191,646 (53.1) | 191,646 (53.1) | 1 |
|  | Male | 169,037 (46.9) | 169,037 (46.9) | 169,037 (46.9) |  |
| SIMD deciles | 1 | 75,728 (21.0) | 75,728 (21.0) | 75,728 (21.0) | 1 |
|  | 2 | 48,939 (13.6) | 48,939 (13.6) | 48,939 (13.6) |  |
|  | 3 | 31,176 (8.6) | 31,176 (8.6) | 31,176 (8.6) |  |
|  | 4 | 28,064 (7.8) | 28,064 (7.8) | 28,064 (7.8) |  |
|  | 5 | 25,100 (7.0) | 25,100 (7.0) | 25,100 (7.0) |  |
|  | 6 | 24,460 (6.8) | 24,460 (6.8) | 24,460 (6.8) |  |
|  | 7 | 21,327 (5.9) | 21,327 (5.9) | 21,327 (5.9) |  |
|  | 8 | 30,432 (8.4) | 30,432 (8.4) | 30,432 (8.4) |  |
|  | 9 | 40,058 (11.1) | 40,058 (11.1) | 40,058 (11.1) |  |
|  | 10 | 35,399 (9.8) | 35,399 (9.8) | 35,399 (9.8) |  |
| MLTCs categories | 0 MLTCs | 176,886 (49.0) | 152052 (42.2) | 140,965 (39.1) | <0.001 |
|  | 1 MLTCs | 52,149 (14.5) | 45540 (12.6) | 41,462 (11.5) |  |
|  | 2 MLTCs | 42,149 (11.7) | 40777 (11.3) | 38,951 (10.8) |  |
|  | 3 MLTCs | 31,560 (8.8) | 34256 (9.5) | 34,435 (9.5) |  |
|  | 4 MLTCs | 22,136 (6.1) | 26834 (7.4) | 28,367 (7.9) |  |
|  | 5+ MLTCs | 35,803 (9.9) | 61224 (17.0) | 76,503 (21.2) |  |
| Total medications in study year - categories | 0 medication | 130,352 (36.1) | 125,314 (34.7) | 124,671 (34.6) | <0.001 |
|  | 1 medication | 17,302 (4.8) | 15,778 (4.4) | 15,223 (4.2) |  |
|  | 2 medications | 18,848 (5.2) | 17,574 (4.9) | 17,441 (4.8) |  |
|  | 3-4 medications | 38,418 (10.7) | 36,848 (10.2) | 36,948 (10.2) |  |
|  | 5+ medications | 155,763 (43.2) | 165,169 (45.8) | 166,400 (46.1) |  |

Supplementary table 10 Sensitivity analysis for patients characteristics in 2016, stratified Scottish Index of Multiple Deprivation (SIMD) deciles. The cohort was restricted to participants who were alive at the end of the study period in 2021. SIMD 1 represents the most deprived population, and SIMD10 corresponds to the most affluent. MLTCs: Multiple long-term conditions, SIMD: Scottish Index of Multiple Deprivation

| Label | Levels | 1 | 2 | 3 | 4 | 5 | 6 | 7 | 8 | 9 | 10 | P |
| --- | --- | --- | --- | --- | --- | --- | --- | --- | --- | --- | --- | --- |
| Total N (%) |  | 75,728 (21.0) | 48,939 (13.6) | 31,176 (8.6) | 28,064 (7.8) | 25,100 (7.0) | 24,460 (6.8) | 21,327 (5.9) | 30,432 (8.4) | 40,058 (11.1) | 35,399 (9.8) |  |
| Age | Mean (SD) | 69.0 (12.1) | 68.5 (11.4) | 68.4 (11.0) | 68.2 (11.0) | 68.3 (11.1) | 67.8 (10.4) | 67.8 (10.1) | 67.4 (10.0) | 67.5 (9.6) | 67.7 (9.6) | <0.001 |
| Sex | Female | 40,236 (53.1) | 26,245 (53.6) | 16,898 (54.2) | 15,026 (53.5) | 13,124 (52.3) | 12,903 (52.8) | 11,505 (53.9) | 15,754 (51.8) | 21,340 (53.3) | 18,615 (52.6) | <0.001 |
|  | Male | 35,492 (46.9) | 22,694 (46.4) | 14,278 (45.8) | 13,038 (46.5) | 11,976 (47.7) | 11,557 (47.2) | 9,822 (46.1) | 14,678 (48.2) | 18,718 (46.7) | 16,784 (47.4) |  |
| MLTCs categories | 0 MLTCs | 37,333 (49.3) | 22,770 (46.5) | 14,324 (45.9) | 13,322 (47.5) | 12,566 (50.1) | 12,277 (50.2) | 9,800 (46.0) | 16,160 (53.1) | 19,835 (49.5) | 18,499 (52.3) | <0.001 |
|  | 1 MLTCs | 8,329 (11.0) | 6,255 (12.8) | 4,311 (13.8) | 4,069 (14.5) | 3,490 (13.9) | 3,772 (15.4) | 3,677 (17.2) | 4,892 (16.1) | 7,172 (17.9) | 6,182 (17.5) |  |
|  | 2 MLTCs | 7,935 (10.5) | 5,591 (11.4) | 3,711 (11.9) | 3,274 (11.7) | 2,920 (11.6) | 2,859 (11.7) | 2,803 (13.1) | 3,578 (11.8) | 5,067 (12.6) | 4,411 (12.5) |  |
|  | 3 MLTCs | 6,666 (8.8) | 4,499 (9.2) | 2,926 (9.4) | 2,633 (9.4) | 2,155 (8.6) | 2,182 (8.9) | 1,894 (8.9) | 2,438 (8.0) | 3,367 (8.4) | 2,800 (7.9) |  |
|  | 4 MLTCs | 5,408 (7.1) | 3,566 (7.3) | 2,140 (6.9) | 1,779 (6.3) | 1,533 (6.1) | 1,335 (5.5) | 1,269 (6.0) | 1,433 (4.7) | 2,079 (5.2) | 1,594 (4.5) |  |
|  | 5+ MLTCs | 10,057 (13.3) | 6,258 (12.8) | 3,764 (12.1) | 2,987 (10.6) | 2,436 (9.7) | 2,035 (8.3) | 1,884 (8.8) | 1,931 (6.3) | 2,538 (6.3) | 1,913 (5.4) |  |
| Total medicines in study year - categories | 0 medication | 30,740 (40.6) | 17,964 (36.7) | 10,751 (34.5) | 9,871 (35.2) | 9,568 (38.1) | 8,883 (36.3) | 6,487 (30.4) | 11,660 (38.3) | 12,705 (31.7) | 11,723 (33.1) | <0.001 |
|  | 1 medication | 2,242 (3.0) | 1,683 (3.4) | 1,296 (4.2) | 1,216 (4.3) | 1,107 (4.4) | 1,296 (5.3) | 1,277 (6.0) | 1,745 (5.7) | 2,767 (6.9) | 2,673 (7.6) |  |
|  | 2 medications | 2,488 (3.3) | 1,990 (4.1) | 1,443 (4.6) | 1,404 (5.0) | 1,209 (4.8) | 1,418 (5.8) | 1,357 (6.4) | 1,845 (6.1) | 2,962 (7.4) | 2,732 (7.7) |  |
|  | 3-4 medications | 5,794 (7.7) | 4,366 (8.9) | 2,963 (9.5) | 3,006 (10.7) | 2,597 (10.3) | 2,774 (11.3) | 2,778 (13.0) | 3,686 (12.1) | 5,453 (13.6) | 5,001 (14.1) |  |
|  | 5+ medications | 34,464 (45.5) | 22,936 (46.9) | 14,723 (47.2) | 12,567 (44.8) | 10,619 (42.3) | 10,089 (41.2) | 9,428 (44.2) | 11,496 (37.8) | 16,171 (40.4) | 13,270 (37.5) |  |

Supplementary table 11 Sensitivity analysis for patients characteristics in 2019, stratified Scottish Index of Multiple Deprivation (SIMD) deciles. The cohort was restricted to participants who were alive at the end of the study period in 2021. SIMD 1 represents the most deprived population, and SIMD10 corresponds to the most affluent. MLTCs: Multiple long-term conditions, SIMD: Scottish Index of Multiple Deprivation

| Label | Levels | 1 | 2 | 3 | 4 | 5 | 6 | 7 | 8 | 9 | 10 | P |
| --- | --- | --- | --- | --- | --- | --- | --- | --- | --- | --- | --- | --- |
| Total N (%) |  | 75,728 (21.0) | 48,939 (13.6) | 31,176 (8.6) | 28,064 (7.8) | 25,100 (7.0) | 24,460 (6.8) | 21,327 (5.9) | 30,432 (8.4) | 40,058 (11.1) | 35,399 (9.8) |  |
| Age | Mean (SD) | 72.0 (12.1) | 71.5 (11.4) | 71.4 (11.0) | 71.2 (11.0) | 71.3 (11.1) | 70.8 (10.4) | 70.8 (10.1) | 70.4 (10.0) | 70.5 (9.6) | 70.7 (9.6) | <0.001 |
| Sex | Female | 40,236 (53.1) | 26,245 (53.6) | 16,898 (54.2) | 15,026 (53.5) | 13,124 (52.3) | 12,903 (52.8) | 11,505 (53.9) | 15,754 (51.8) | 2,1340 (53.3) | 18,615 (52.6) | <0.001 |
|  | Male | 35,492 (46.9) | 22,694 (46.4) | 14,278 (45.8) | 13,038 (46.5) | 11,976 (47.7) | 11,557 (47.2) | 9,822 (46.1) | 14,678 (48.2) | 18,718 (46.7) | 16,784 (47.4) |  |
| MLTCs categories | 0 MLTCs | 33,018 (43.6) | 19,862 (40.6) | 12,271 (39.4) | 11,429 (40.7) | 10,926 (43.5) | 10,506 (42.9) | 8,096 (38.0) | 13,953 (45.8) | 16,442 (41.0) | 15,549 (43.9) | <0.001 |
|  | 1 MLTCs | 6,773 (8.9) | 5,011 (10.2) | 3,598 (11.5) | 3,501 (12.5) | 3,024 (12.0) | 3,317 (13.6) | 3,358 (15.7) | 4,414 (14.5) | 6,719 (16.8) | 5,825 (16.5) |  |
|  | 2 MLTCs | 7,040 (9.3) | 5,112 (10.4) | 3,455 (11.1) | 3,146 (11.2) | 2,767 (11.0) | 2,830 (11.6) | 2,795 (13.1) | 3,720 (12.2) | 5,200 (13.0) | 4,712 (13.3) |  |
|  | 3 MLTCs | 6,701 (8.8) | 4,689 (9.6) | 3,051 (9.8) | 2,778 (9.9) | 2,327 (9.3) | 2,395 (9.8) | 2,116 (9.9) | 2,796 (9.2) | 4,072 (10.2) | 3,331 (9.4) |  |
|  | 4 MLTCs | 5,899 (7.8) | 3,949 (8.1) | 2,517 (8.1) | 2,159 (7.7) | 1,867 (7.4) | 1,752 (7.2) | 1,672 (7.8) | 1,927 (6.3) | 2,795 (7.0) | 2,297 (6.5) |  |
|  | 5+ MLTCs | 16,297 (21.5) | 10,316 (21.1) | 6,284 (20.2) | 5,051 (18.0) | 4,189 (16.7) | 3,660 (15.0) | 3,290 (15.4) | 3,622 (11.9) | 4,830 (12.1) | 3,685 (10.4) |  |
| Total medicines in study year - categories | 0 medication | 30,077 (39.7) | 17,513 (35.8) | 10,367 (33.3) | 9,419 (33.6) | 9,176 (36.6) | 8,537 (34.9) | 6,105 (28.6) | 11,160 (36.7) | 11,888 (29.7) | 11,072 (31.3) | <0.001 |
|  | 1 medication | 1,913 (2.5) | 1,473 (3.0) | 1,098 (3.5) | 1,144 (4.1) | 1,044 (4.2) | 1,190 (4.9) | 1,202 (5.6) | 1,689 (5.6) | 2,497 (6.2) | 2,528 (7.1) |  |
|  | 2 medications | 2,329 (3.1) | 1,738 (3.6) | 1,329 (4.3) | 1,303 (4.6) | 1,201 (4.8) | 1,253 (5.1) | 1,280 (6.0) | 1,838 (6.0) | 2,773 (6.9) | 2,530 (7.1) |  |
|  | 3-4 medications | 5,349 (7.1) | 4,134 (8.4) | 2,944 (9.4) | 2,806 (10.0) | 2,424 (9.7) | 2,732 (11.2) | 2,642 (12.4) | 3,519 (11.6) | 5,439 (13.6) | 4,859 (13.7) |  |
|  | 5+ medications | 36,060 (47.6) | 24,081 (49.2) | 15,438 (49.5) | 13,392 (47.7) | 11,255 (44.8) | 10,748 (43.9) | 10,098 (47.3) | 12,226 (40.2) | 17,461 (43.6) | 14,410 (40.7) |  |

Supplementary table 12 Sensitivity analysis for patients characteristics in 2021, stratified Scottish Index of Multiple Deprivation (SIMD) deciles. The cohort was restricted to participants who were alive at the end of the study period in 2021. SIMD 1 represents the most deprived population, and SIMD10 corresponds to the most affluent. MLTCs: Multiple long-term conditions, SIMD: Scottish Index of Multiple Deprivation

| Label | Levels | 1 | 2 | 3 | 4 | 5 | 6 | 7 | 8 | 9 | 10 | P |
| --- | --- | --- | --- | --- | --- | --- | --- | --- | --- | --- | --- | --- |
| Total N (%) |  | 75,728 (21.0) | 48,939 (13.6) | 31,176 (8.6) | 28,064 (7.8) | 25,100 (7.0) | 24,460 (6.8) | 21,327 (5.9) | 30,432 (8.4) | 40,058 (11.1) | 35,399 (9.8) |  |
| Age | Mean (SD) | 74.0 (12.1) | 73.5 (11.4) | 73.4 (11.0) | 73.2 (11.0) | 73.3 (11.1) | 72.8 (10.4) | 72.8 (10.1) | 72.4 (10.0) | 72.5 (9.6) | 72.7 (9.6) | <0.001 |
| Sex | Female | 40,236 (53.1) | 26,245 (53.6) | 16,898 (54.2) | 15,026 (53.5) | 13,124 (52.3) | 12,903 (52.8) | 11,505 (53.9) | 15,754 (51.8) | 21,340 (53.3) | 18,615 (52.6) | <0.001 |
|  | Male | 35,492 (46.9) | 22,694 (46.4) | 14,278 (45.8) | 13,038 (46.5) | 11,976 (47.7) | 11,557 (47.2) | 9,822 (46.1) | 14,678 (48.2) | 18,718 (46.7) | 16,784 (47.4) |  |
| MLTCs categories | 0 MLTCs | 31,158 (41.1) | 18,584 (38.0) | 11,344 (36.4) | 10,564 (37.6) | 10,162 (40.5) | 9,714 (39.7) | 7,301 (34.2) | 12,919 (42.5) | 14,946 (37.3) | 14,273 (40.3) | <0.001 |
|  | 1 MLTCs | 6,011 (7.9) | 4,468 (9.1) | 3,226 (10.3) | 3,220 (11.5) | 2,711 (10.8) | 3,034 (12.4) | 3,030 (14.2) | 4,132 (13.6) | 6,176 (15.4) | 5,454 (15.4) |  |
|  | 2 MLTCs | 6,500 (8.6) | 4,686 (9.6) | 3,266 (10.5) | 2,971 (10.6) | 2,619 (10.4) | 2,694 (11.0) | 2,688 (12.6) | 3,621 (11.9) | 5,218 (13.0) | 4,688 (13.2) |  |
|  | 3 MLTCs | 6,461 (8.5) | 4,516 (9.2) | 3,000 (9.6) | 2,748 (9.8) | 2,313 (9.2) | 2,419 (9.9) | 2,260 (10.6) | 2,935 (9.6) | 4,208 (10.5) | 3,575 (10.1) |  |
|  | 4 MLTCs | 5,915 (7.8) | 3,965 (8.1) | 2,628 (8.4) | 2,281 (8.1) | 2,036 (8.1) | 1,953 (8.0) | 1,796 (8.4) | 2,101 (6.9) | 3,161 (7.9) | 2,531 (7.1) |  |
|  | 5+ MLTCs | 19,683 (26.0) | 12,720 (26.0) | 7,712 (24.7) | 6,280 (22.4) | 5,259 (21.0) | 4,646 (19.0) | 4,252 (19.9) | 4,724 (15.5) | 6,349 (15.8) | 4,878 (13.8) |  |
| Total medicines in study year - categories | 0 medication | 30,194 (39.9) | 17,453 (35.7) | 10,273 (33.0) | 9,418 (33.6) | 9,144 (36.4) | 8,435 (34.5) | 5,949 (27.9) | 11,053 (36.3) | 11,775 (29.4) | 10,977 (31.0) | <0.001 |
|  | 1 medication | 1,784 (2.4) | 1,422 (2.9) | 1,124 (3.6) | 1,126 (4.0) | 975 (3.9) | 1,122 (4.6) | 1,165 (5.5) | 1,589 (5.2) | 2,490 (6.2) | 2,426 (6.9) |  |
|  | 2 medications | 2,267 (3.0) | 1,748 (3.6) | 1,287 (4.1) | 1,277 (4.6) | 1,105 (4.4) | 1,282 (5.2) | 1,367 (6.4) | 1,800 (5.9) | 2,733 (6.8) | 2,575 (7.3) |  |
|  | 3-4 medications | 5,413 (7.1) | 4,138 (8.5) | 2,851 (9.1) | 2,809 (10.0) | 2,497 (9.9) | 2,676 (10.9) | 2,631 (12.3) | 3,586 (11.8) | 5,388 (13.5) | 4,959 (14.0) |  |
|  | 5+ medications | 36,070 (47.6) | 24,178 (49.4) | 15,641 (50.2) | 13,434 (47.9) | 11,379 (45.3) | 10,945 (44.7) | 10,215 (47.9) | 12,404 (40.8) | 17,672 (44.1) | 14,462 (40.9) |  |

Supplementary table 13 Sensitivty analysis for the comparison of SIMD10 and SIMD1 risk ratios across multiple long-term condition (MLTCs) categories in 2016 and 2021, using 0 or 1 MLTCs as reference groups. The cohort was restricted to participants who were alive at the end of the study period in 2021. RR: risk ration, SIMD: Scottish Index of Multiple Deprivation.

|  | Categories of MLTCs number | 2016 SIMD10 vs SIMD1  RR (95% CI) | 2021 SIMD10 vs SIMD1  RR (95% CI) | SIMD1 2021 vs 2016  RR (95% CI) | SIMD10 2021 vs 2016  RR (95% CI) |
| --- | --- | --- | --- | --- | --- |
| Using zero MLTCs as the reference category | 0 MLTCs | 1 | 1 | 1 | 1 |
|  | 1 MLTCs | 1.5 [1.47 - 1.52]; p<0.001 | 1.98 [1.95 - 2.01]; p<0.001 | 0.86 [0.85 - 0.88]; p<0.001 | 1.14 [1.13 - 1.16]; p<0.001 |
|  | 2 MLTCs | 1.12 [1.1 - 1.15]; p<0.001 | 1.57 [1.54 - 1.61]; p<0.001 | 0.98 [0.97 - 0.99]; p=0.31 | 1.38 [1.37 - 1.38]; p<0.001 |
|  | 3 MLTCs | 0.85 [0.82 - 0.87]; p<0.001 | 1.21 [1.18 - 1.24]; p<0.001 | 1.16 [1.15 - 1.17]; p<0.001 | 1.65 [1.63 - 1.68]; p<0.001 |
|  | 4 MLTCs | 0.59 [0.57 - 0.62]; p<0.001 | 0.93 [0.9 - 0.96]; p=0.008 | 1.31 [1.3 - 1.32]; p<0.001 | 2.06 [2 - 2.12]; p<0.001 |
|  | 5+ MLTCs | 0.38 [0.37 - 0.4]; p<0.001 | 0.54 [0.53 - 0.56]; p<0.001 | 2.35 [2.31 - 2.38]; p<0.001 | 3.3 [3.19 - 3.42]; p<0.001 |
| Using zero MLTCs as the reference category  (Cumulative) | 0 MLTCs | 1 | 1 | 1 | 1 |
|  | 1+ MLTCs | 0.89 [0.87 - 0.9]; p<0.001 | 1.03 [1.02 - 1.05]; p=0.009 | 1.39 [1.39 - 1.39]; p<0.001 | 1.62 [1.61 - 1.63]; p<0.001 |
|  | 2+ MLTCs | 0.72 [0.71 - 0.73]; p<0.001 | 0.89 [0.87 - 0.9]; p<0.001 | 1.54 [1.53 - 1.54]; p<0.001 | 1.9 [1.88 - 1.91]; p<0.001 |
|  | 3+ MLTCs | 0.58 [0.56 - 0.59]; p<0.001 | 0.75 [0.73 - 0.76]; p<0.001 | 1.74 [1.73 - 1.75]; p<0.001 | 2.26 [2.23 - 2.29]; p<0.001 |
|  | 4+ MLTCs | 0.46 [0.44 - 0.47]; p<0.001 | 0.63 [0.62 - 0.65]; p<0.001 | 1.98 [1.97 - 2]; p<0.001 | 2.74 [2.68 - 2.8]; p<0.001 |
|  | 5+ MLTCs | 0.38 [0.37 - 0.4]; p<0.001 | 0.54 [0.53 - 0.56]; p<0.001 | 2.35 [2.31 - 2.38]; p<0.001 | 3.3 [3.19 - 3.42]; p<0.001 |
| Using one MLTCs as the reference category | 1 MLTCs | 1 | 1 | 1 | 1 |
|  | 2 MLTCs | 0.75 [0.73 - 0.77]; p<0.001 | 0.79 [0.78 - 0.81]; p<0.001 | 1.14 [1.12 - 1.15]; p<0.001 | 1.2 [1.2 - 1.21]; p<0.001 |
|  | 3 MLTCs | 0.57 [0.55 - 0.58]; p<0.001 | 0.61 [0.6 - 0.62]; p<0.001 | 1.34 [1.32 - 1.36]; p<0.001 | 1.45 [1.43 - 1.47]; p<0.001 |
|  | 4 MLTCs | 0.4 [0.38 - 0.41]; p<0.001 | 0.47 [0.46 - 0.49]; p<0.001 | 1.52 [1.5 - 1.53]; p<0.001 | 1.8 [1.75 - 1.85]; p<0.001 |
|  | 5+ MLTCs | 0.26 [0.25 - 0.27]; p<0.001 | 0.27 [0.27 - 0.28]; p<0.001 | 2.71 [2.7 - 2.72]; p<0.001 | 2.89 [2.79 - 2.99]; p<0.001 |
| Using one MLTCs as the reference category  (Cumulative) | 1 MLTCs | 1 | 1 | 1 | 1 |
|  | 2+ MLTCs | 0.48 [0.47 - 0.49]; p<0.001 | 0.45 [0.44 - 0.45]; p<0.001 | 1.78 [1.76 - 1.8]; p<0.001 | 1.66 [1.65 - 1.67]; p<0.001 |
|  | 3+ MLTCs | 0.38 [0.37 - 0.39]; p<0.001 | 0.38 [0.37 - 0.38]; p<0.001 | 2.01 [1.98 - 2.03]; p<0.001 | 1.97 [1.95 - 2]; p<0.001 |
|  | 4+ MLTCs | 0.31 [0.3 - 0.32]; p<0.001 | 0.32 [0.31 - 0.33]; p<0.001 | 2.29 [2.27 - 2.31]; p<0.001 | 2.39 [2.34 - 2.45]; p<0.001 |
|  | 5+ MLTCs | 0.17 [0.16 - 0.17]; p<0.001 | 0.27 [0.27 - 0.28]; p<0.001 | 1.77 [1.75 - 1.79]; p<0.001 | 2.89 [2.79 - 2.99]; p<0.001 |

Supplementary table 14 Sensitivty analysis for the comparison of SIMD10 and SIMD1 risk ratios across multiple medication number categories in 2016 and 2021, using 0 or 1 MLTCs as reference groups. The cohort was restricted to participants who were alive at the end of the study period in 2021. RR: risk ration, SIMD: Scottish Index of Multiple Deprivation.

|  | Categories of medication number | 2016 SIMD10 vs SIMD1  RR (95% CI) | 2021 SIMD10 vs SIMD1  RR (95% CI) | SIMD1 2021 vs 2016  RR (95% CI) | SIMD10 2021 vs 2016  RR (95% CI) |
| --- | --- | --- | --- | --- | --- |
| Using zero medication as the reference category | 0 medication | 1 | 1 | 1 | 1 |
|  | 1 medication | 3.13 [3.1 - 3.15]; p<0.001 | 3.74 [3.67 - 3.81]; p<0.001 | 0.81 [0.79 - 0.83]; p<0.001 | 0.97 [0.96 - 0.98]; p=0.315 |
|  | 2 medications | 2.88 [2.86 - 2.9]; p<0.001 | 3.12 [3.11 - 3.14]; p<0.001 | 0.93 [0.92 - 0.94]; p=0.013 | 1.01 [1 - 1.02]; p=0.83 |
|  | 3-4 medications | 2.26 [2.22 - 2.3]; p<0.001 | 2.52 [2.48 - 2.56]; p<0.001 | 0.95 [0.94 - 0.96]; p=0.015 | 1.06 [1.05 - 1.06]; p=0.017 |
|  | 5+ medications | 1.01 [0.99 - 1.03]; p=0.519 | 1.1 [1.08 - 1.12]; p<0.001 | 1.07 [1.06 - 1.07]; p<0.001 | 1.16 [1.16 - 1.17]; p<0.001 |
| Using zero medication as the reference category  (Cumulative) | 0 medication | 1 | 1 | 1 | 1 |
|  | 2+ medications | 1.29 [1.27 - 1.31]; p<0.001 | 1.38 [1.36 - 1.41]; p<0.001 | 1.04 [1.04 - 1.04]; p<0.001 | 1.12 [1.11 - 1.12]; p<0.001 |
|  | 3+ medications | 1.19 [1.17 - 1.21]; p<0.001 | 1.29 [1.26 - 1.31]; p<0.001 | 1.05 [1.05 - 1.05]; p<0.001 | 1.14 [1.13 - 1.14]; p<0.001 |
| Using one medication as the reference category | 1 medication | 1 | 1 | 1 | 1 |
|  | 2 medications | 0.92 [0.9 - 0.94]; p=0.039 | 0.84 [0.81 - 0.86]; p<0.001 | 1.15 [1.12 - 1.17]; p=0.002 | 1.04 [1.02 - 1.05]; p=0.336 |
|  | 3-4 medications | 0.72 [0.71 - 0.73]; p<0.001 | 0.67 [0.66 - 0.69]; p<0.001 | 1.17 [1.15 - 1.2]; p<0.001 | 1.09 [1.08 - 1.11]; p=0.01 |
|  | 5+ medications | 0.32 [0.32 - 0.33]; p<0.001 | 0.29 [0.29 - 0.3]; p<0.001 | 1.32 [1.29 - 1.34]; p<0.001 | 1.2 [1.19 - 1.21]; p<0.001 |
| Using one medication as the reference category  (Cumulative) | 1 medications | 1 | 1 | 1 | 1 |
|  | 2+ medications | 0.41 [0.41 - 0.42]; p<0.001 | 0.37 [0.36 - 0.38]; p<0.001 | 1.29 [1.26 - 1.31]; p<0.001 | 1.15 [1.14 - 1.17]; p<0.001 |
|  | 3+ medications | 0.38 [0.38 - 0.39]; p<0.001 | 0.34 [0.34 - 0.35]; p<0.001 | 1.29 [1.27 - 1.32]; p<0.001 | 1.17 [1.16 - 1.18]; p<0.001 |

Supplementary table 15 Sensitivity analysis results from the Zero-Inflated Negative Binomial (ZINB) model analyzing count and zero-inflation components of MLTCs across 10 SIMD deciles in 2016, 2019, and 2021. The cohort for each year was restricted to participants who were alive at the end of the study period in 2021. The count component columns present incidence rate ratios (IRR) for MLTCs counts, using a negative binomial distribution, while the zero-inflation component columns present odds ratios (OR) from logistic regression for membership in the zero MLTCs group. SIMD 1 represents the most deprived population, and SIMD 10 corresponds to the most affluent. IRR: incidence rate ratio, OR: odd ratio, SIMD: Scottish Index of Multiple Deprivation.

| SIMD | Count component - 2016 IRR (95% CI) | Count component - 2019 IRR (95% CI) | Count component - 2021 IRR (95% CI) | Zero inflation component - 2016 OR (95% CI) | Zero inflation component - 2019 OR (95% CI) | Zero inflation component - 2021 OR (95% CI) |
| --- | --- | --- | --- | --- | --- | --- |
| SIMD 1 | 1 | 1 | 1 | 1 | 1 | 1 |
| SIMD 2 | 0.92 [0.91 - 0.94] ; P value <0.001 | 0.93 [0.92 - 0.94] ; P value <0.001 | 0.94 [0.93 - 0.95] ; P value <0.001 | 0.82 [0.8 - 0.85] ; P value <0.001 | 0.83 [0.81 - 0.85] ; P value <0.001 | 0.83 [0.81 - 0.86] ; P value <0.001 |
| SIMD 3 | 0.86 [0.85 - 0.88] ; P value <0.001 | 0.88 [0.86 - 0.89] ; P value <0.001 | 0.88 [0.87 - 0.89] ; P value <0.001 | 0.76 [0.73 - 0.79] ; P value <0.001 | 0.75 [0.72 - 0.77] ; P value <0.001 | 0.74 [0.72 - 0.77] ; P value <0.001 |
| SIMD 4 | 0.82 [0.81 - 0.84] ; P value <0.001 | 0.83 [0.82 - 0.84] ; P value <0.001 | 0.84 [0.83 - 0.85] ; P value <0.001 | 0.79 [0.76 - 0.82] ; P value <0.001 | 0.78 [0.75 - 0.81] ; P value <0.001 | 0.77 [0.75 - 0.8] ; P value <0.001 |
| SIMD 5 | 0.79 [0.78 - 0.81] ; P value <0.001 | 0.8 [0.79 - 0.82] ; P value <0.001 | 0.82 [0.8 - 0.83] ; P value <0.001 | 0.87 [0.83 - 0.9] ; P value <0.001 | 0.87 [0.84 - 0.9] ; P value <0.001 | 0.87 [0.84 - 0.9] ; P value <0.001 |
| SIMD 6 | 0.72 [0.71 - 0.74] ; P value <0.001 | 0.74 [0.73 - 0.75] ; P value <0.001 | 0.75 [0.74 - 0.76] ; P value <0.001 | 0.81 [0.78 - 0.85] ; P value <0.001 | 0.8 [0.77 - 0.83] ; P value <0.001 | 0.8 [0.77 - 0.83] ; P value <0.001 |
| SIMD 7 | 0.7 [0.68 - 0.71] ; P value <0.001 | 0.71 [0.7 - 0.72] ; P value <0.001 | 0.72 [0.71 - 0.74] ; P value <0.001 | 0.6 [0.57 - 0.63] ; P value <0.001 | 0.57 [0.54 - 0.59] ; P value <0.001 | 0.56 [0.53 - 0.58] ; P value <0.001 |
| SIMD 8 | 0.64 [0.63 - 0.65] ; P value <0.001 | 0.66 [0.64 - 0.67] ; P value <0.001 | 0.67 [0.66 - 0.67] ; P value <0.001 | 0.85 [0.81 - 0.88] ; P value <0.001 | 0.85 [0.82 - 0.88] ; P value <0.001 | 0.85 [0.82 - 0.88] ; P value <0.001 |
| SIMD 9 | 0.61 [0.6 - 0.62] ; P value <0.001 | 0.62 [0.61 - 0.63] ; P value <0.001 | 0.63 [0.63 - 0.64] ; P value <0.001 | 0.63 [0.6 - 0.65] ; P value <0.001 | 0.59 [0.57 - 0.62] ; P value <0.001 | 0.59 [0.57 - 0.61] ; P value <0.001 |
| SIMD 10 | 0.56 [0.55 - 0.58] ; P value <0.001 | 0.58 [0.57 - 0.59] ; P value <0.001 | 0.59 [0.58 - 0.6] ; P value <0.001 | 0.68 [0.65 - 0.71] ; P value <0.001 | 0.65 [0.63 - 0.68] ; P value <0.001 | 0.65 [0.63 - 0.68] ; P value <0.001 |

Supplementary table 16 Sensitivity analysis results from the Zero-Inflated Negative Binomial (ZINB) model analyzing count and zero-inflation components of total medications across 10 SIMD deciles in 2016, 2019, and 2021. The cohort for each year was restricted to participants who were alive at the end of the study period in 2021. The count component columns present incidence rate ratios (IRR) for total medication counts, using a negative binomial distribution, while the zero-inflation component columns present odds ratios (OR) from logistic regression for membership in the zero total medication group. SIMD 1 represents the most deprived population, and SIMD 10 corresponds to the most affluent. IRR: incidence rate ratio, OR: odd ratio, SIMD: Scottish Index of Multiple Deprivation.

| SIMD | Count component - 2016 IRR (95% CI) | Count component - 2019 IRR (95% CI) | Count component - 2021 IRR (95% CI) | Zero inflation component - 2016 OR (95% CI) | Zero inflation component - 2019 OR (95% CI) | Zero inflation component - 2021 OR (95% CI) |
| --- | --- | --- | --- | --- | --- | --- |
| SIMD 1 | 1 | 1 | 1 | 1 | 1 | 1 |
| SIMD 2 | 0.99 [0.98 - 1] ; P value =0.002 | 0.99 [0.99 - 1] ; P value =0.011 | 0.99 [0.98 - 1] ; P value <0.001 | 0.87 [0.82 - 0.93] ; P value <0.001 | 0.87 [0.81 - 0.93] ; P value <0.001 | 0.84 [0.78 - 0.9] ; P value <0.001 |
| SIMD 3 | 0.98 [0.97 - 0.99] ; P value <0.001 | 0.98 [0.98 - 0.99] ; P value <0.001 | 0.98 [0.98 - 0.99] ; P value <0.001 | 0.75 [0.7 - 0.81] ; P value <0.001 | 0.72 [0.66 - 0.77] ; P value <0.001 | 0.71 [0.65 - 0.77] ; P value <0.001 |
| SIMD 4 | 0.97 [0.96 - 0.97] ; P value <0.001 | 0.98 [0.97 - 0.98] ; P value <0.001 | 0.97 [0.96 - 0.98] ; P value <0.001 | 0.73 [0.68 - 0.79] ; P value <0.001 | 0.65 [0.6 - 0.71] ; P value <0.001 | 0.67 [0.62 - 0.73] ; P value <0.001 |
| SIMD 5 | 0.96 [0.95 - 0.97] ; P value <0.001 | 0.97 [0.96 - 0.98] ; P value <0.001 | 0.98 [0.97 - 0.98] ; P value <0.001 | 0.8 [0.74 - 0.86] ; P value <0.001 | 0.73 [0.68 - 0.8] ; P value <0.001 | 0.72 [0.66 - 0.78] ; P value <0.001 |
| SIMD 6 | 0.95 [0.94 - 0.96] ; P value <0.001 | 0.96 [0.95 - 0.96] ; P value <0.001 | 0.96 [0.95 - 0.97] ; P value <0.001 | 0.66 [0.61 - 0.71] ; P value <0.001 | 0.63 [0.58 - 0.69] ; P value <0.001 | 0.6 [0.55 - 0.66] ; P value <0.001 |
| SIMD 7 | 0.94 [0.93 - 0.95] ; P value <0.001 | 0.95 [0.94 - 0.96] ; P value <0.001 | 0.95 [0.94 - 0.96] ; P value <0.001 | 0.52 [0.48 - 0.56] ; P value <0.001 | 0.49 [0.45 - 0.53] ; P value <0.001 | 0.45 [0.41 - 0.49] ; P value <0.001 |
| SIMD 8 | 0.93 [0.92 - 0.94] ; P value <0.001 | 0.94 [0.93 - 0.94] ; P value <0.001 | 0.95 [0.94 - 0.95] ; P value <0.001 | 0.69 [0.64 - 0.73] ; P value <0.001 | 0.66 [0.61 - 0.71] ; P value <0.001 | 0.63 [0.59 - 0.68] ; P value <0.001 |
| SIMD 9 | 0.92 [0.91 - 0.92] ; P value <0.001 | 0.93 [0.93 - 0.94] ; P value <0.001 | 0.93 [0.92 - 0.94] ; P value <0.001 | 0.5 [0.47 - 0.53] ; P value <0.001 | 0.49 [0.46 - 0.53] ; P value <0.001 | 0.46 [0.43 - 0.5] ; P value <0.001 |
| SIMD 10 | 0.89 [0.89 - 0.9] ; P value <0.001 | 0.91 [0.9 - 0.92] ; P value <0.001 | 0.91 [0.9 - 0.91] ; P value <0.001 | 0.5 [0.47 - 0.53] ; P value <0.001 | 0.47 [0.44 - 0.51] ; P value <0.001 | 0.44 [0.41 - 0.47] ; P value <0.001 |

# Supplementary Figures


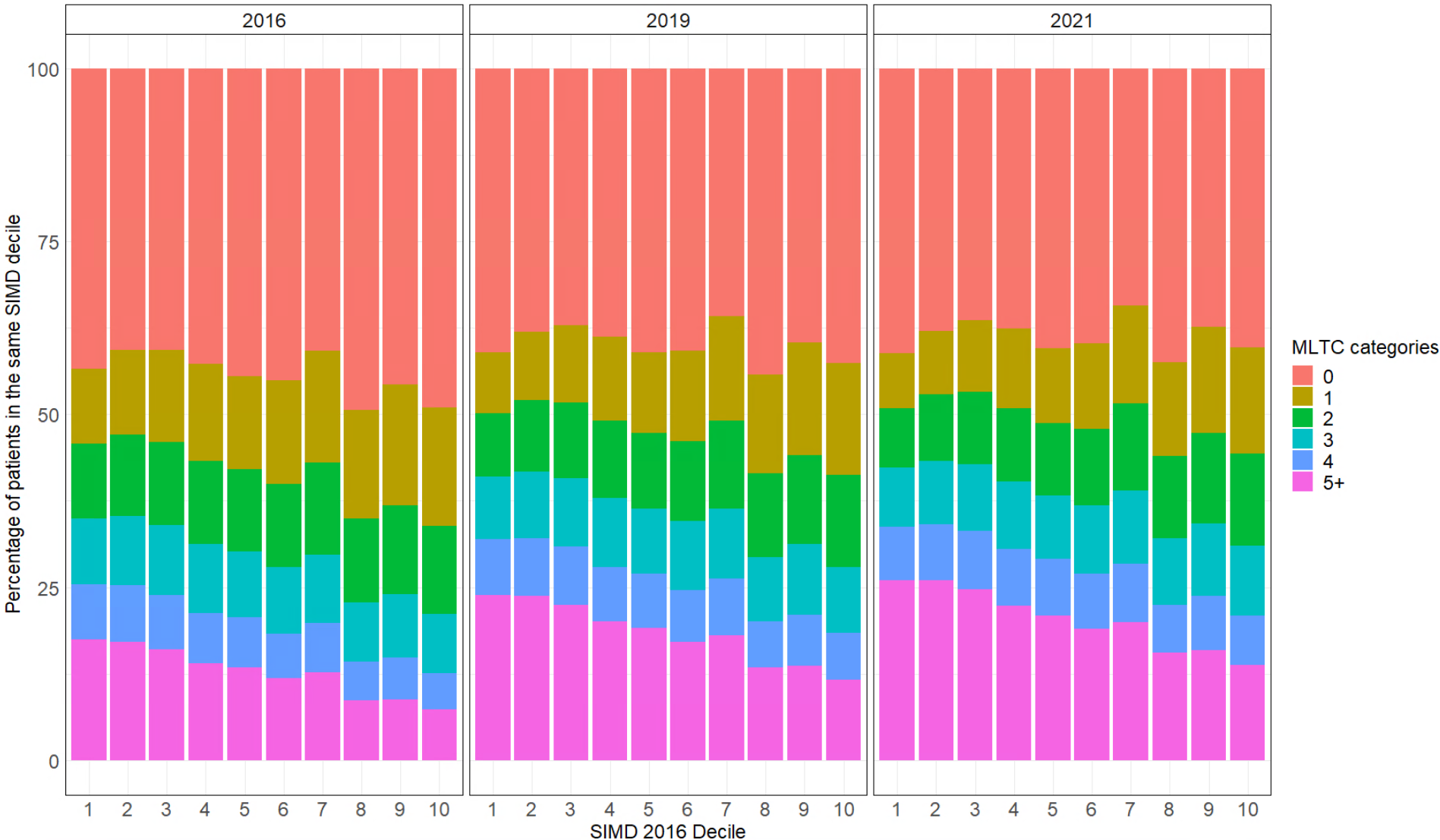


Supplementary figure 1 Stacked bar chart showing the percentage of patients belonging in each MLTCs category, across the 10 SIMD deciles. SIMD 1 represents the most deprived population, and SIMD 10 corresponds to the most affluent. Between 2016 and 2021, a clear socioeconomic gradient existed for severe MLTCs categories (3+ MLTCs), with higher rates in deprived populations, highlighting persistent disparities in complex health burdens among lower socioeconomic groups. MLTCs: multiple long-term conditions, SIMD: Scottish Index of Multiple Deprivation


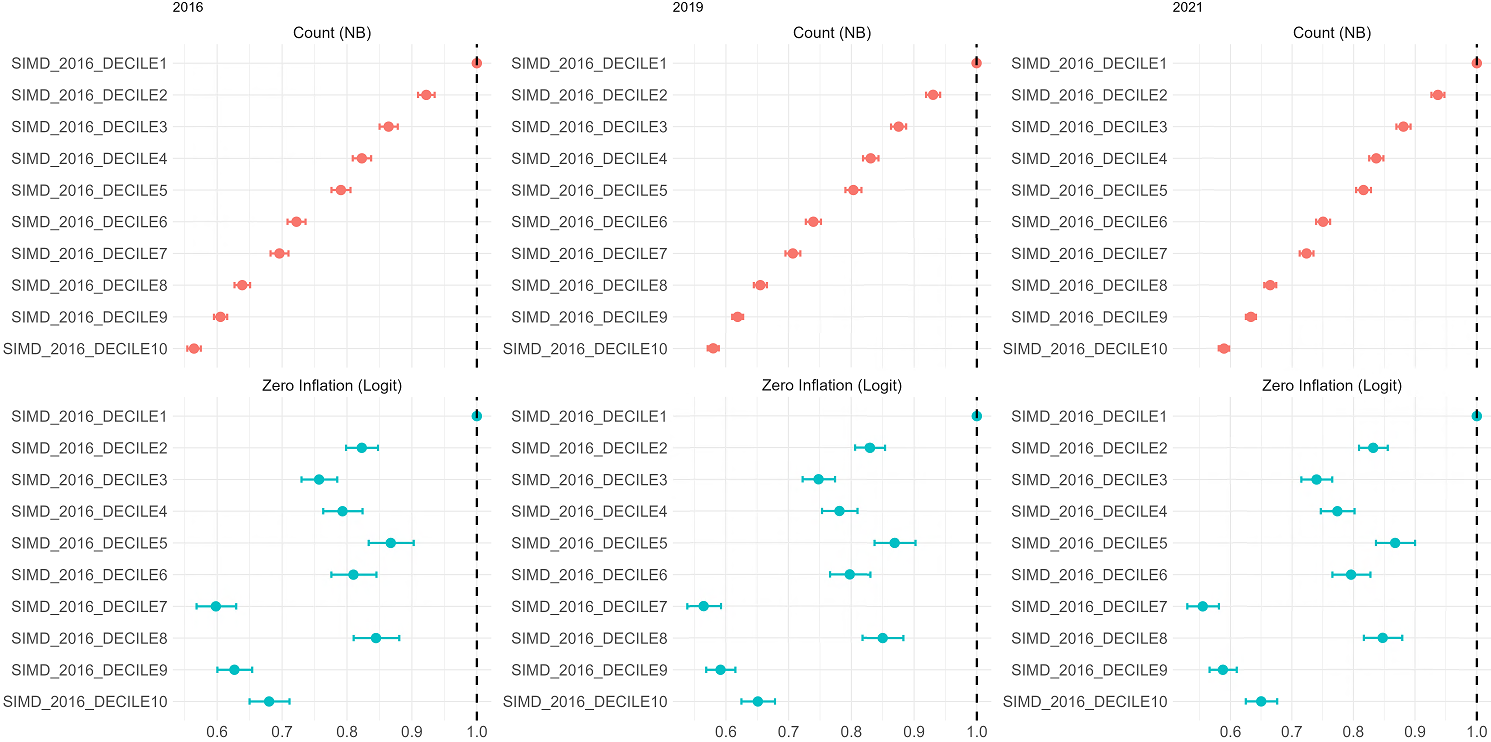


Supplementary figure 2 Forest plot showing sensitivity analysis results from the Zero-Inflated Negative Binomial (ZINB) model analyzing count and zero-inflation components of the number of MLTCs across 10 SIMD deciles in 2016, 2019, and 2021. The cohort for each year was restricted to participants who were alive at the end of the study period in 2021. The count component presents incidence rate ratios (IRR) for MLTCs counts, using a negative binomial distribution, while the zero-inflation component presents odds ratios (OR) from logistic regression for membership in the zero MLTCs group. SIMD 1 represents the most deprived population, and SIMD 10 corresponds to the most affluent. IRR: incidence rate ratio, OR: odd ratio, SIMD: Scottish Index of Multiple Deprivation.


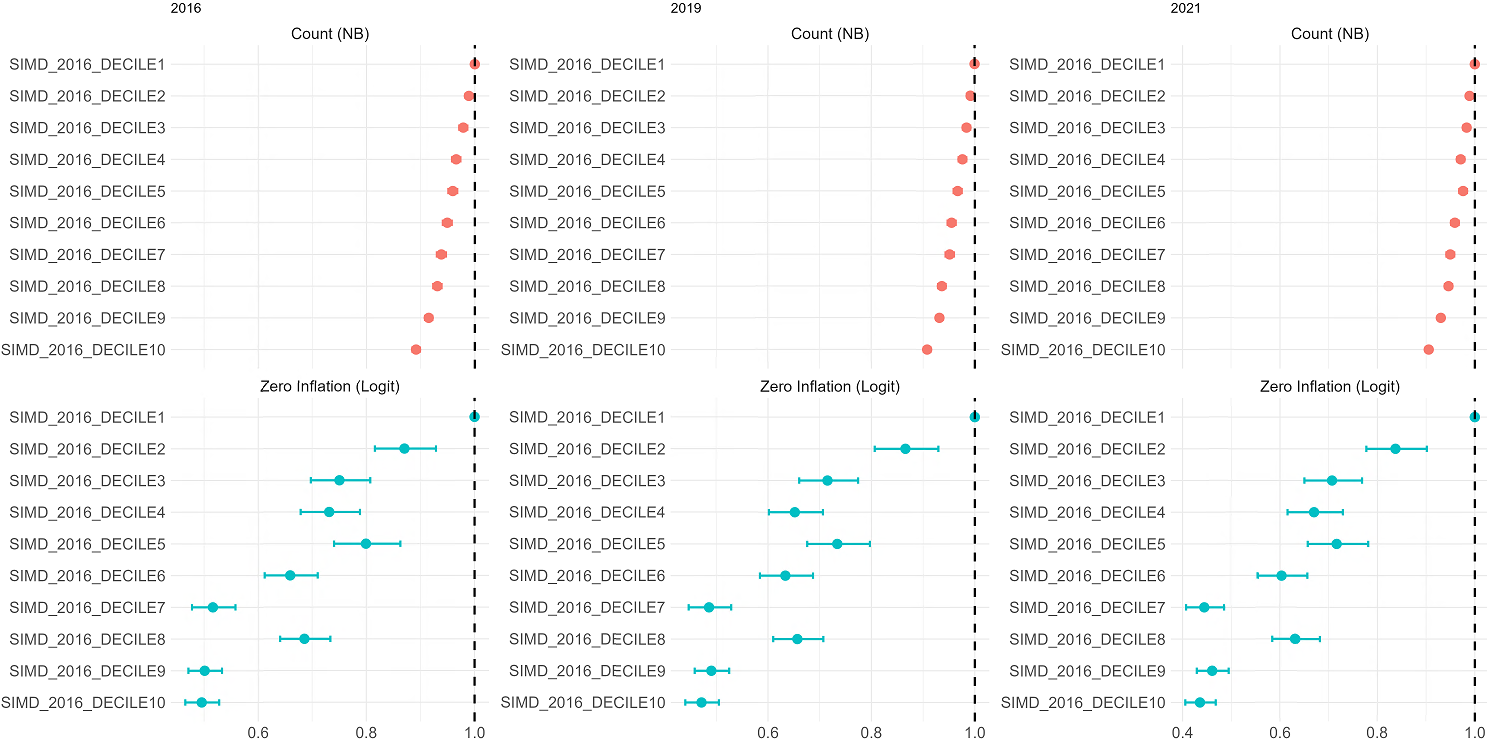


Supplementary figure 3 Forest plot showing sensitivity analysis results from the Zero-Inflated Negative Binomial (ZINB) model analyzing count and zero-inflation components of the number of total medications across 10 SIMD deciles in 2016, 2019, and 2021. The cohort for each year was restricted to participants who were alive at the end of the study period in 2021. The count component presents incidence rate ratios (IRR) for total medication counts, using a negative binomial distribution, while the zero-inflation component presents odds ratios (OR) from logistic regression for membership in the zero total medications group. SIMD 1 represents the most deprived population, and SIMD 10 corresponds to the most affluent. SIMD: Scottish Index of Multiple Deprivation
